# The association of clinical phenotypes to known AD/FTD genetic risk loci and their inter-relationship

**DOI:** 10.1371/journal.pone.0241552

**Published:** 2020-11-05

**Authors:** Qingqin S. Li, Chao Tian, The 23andMe Research Team, David Hinds, Guy R. Seabrook

**Affiliations:** 1 Janssen Research & Development, LLC, Titusville, NJ, United States of America; 2 23andMe, Inc., Mountain View, CA, United States of America; 3 Johnson & Johnson Innovation, South San Francisco, CA, United States of America; Institut de Biomedicina de València-CSIC, SPAIN

## Abstract

To elucidate how variants in genetic risk loci previously implicated in Alzheimer’s Disease (AD) and/or frontotemporal dementia (FTD) contribute to expression of disease phenotypes, a phenome-wide association study was performed in two waves. In the first wave, we explored clinical traits associated with thirteen genetic variants previously reported to be linked to disease risk using both the 23andMe and UKB cohorts. We tested 30 additional AD variants in UKB cohort only in the second wave. *APOE* variants defining **ε**2/**ε**3/**ε**4 alleles and rs646776 were identified to be significantly associated with metabolic/cardiovascular and longevity traits. *APOE* variants were also significantly associated with neurological traits. *ABI3* variant rs28394864 was significantly associated with cardiovascular (e.g. (hypertension, ischemic heart disease, coronary atherosclerosis, angina) and immune-related trait asthma. Both *APOE* variants and *CLU* variant were significantly associated with nearsightedness. *HLA- DRB1* variant was associated with diseases with immune-related traits. Additionally, variants from 10+ AD genes (*BZRAP1-AS1*, *ADAMTS4*, *ADAM10*, *APH1B*, *SCIMP*, *ABI3*, *SPPL2A*, *ZNF232*, *GRN*, *CD2AP*, *and CD33*) were associated with hematological measurements such as white blood cell (leukocyte) count, monocyte count, neutrophill count, platelet count, and/or mean platelet (thrombocyte) volume (an autoimmune disease biomarker). Many of these genes are expressed specifically in microglia. The associations of *ABI3* variant with cardiovascular and immune-related traits are one of the novel findings from this study. Taken together, it is evidenced that at least some AD and FTD variants are associated with multiple clinical phenotypes and not just dementia. These findings were discussed in the context of causal relationship versus pleiotropy via Mendelian randomization analysis.

## Introduction

Genome Wide Association Study (GWAS) is a powerful approach in identifying genetic risk loci. However, the functional elucidation on how a risk locus is related to a disease still requires much functional genomics follow up. Understanding the phenotypic spectrum associated with genetic risk variants from the GWAS signals will shed light in the understanding of disease etiology of the main disease trait of interest (i.e. dementia in this study).

Dementia refers to conditions of memory loss and other cognitive decline serious enough to interfere with daily life. Alzheimer's disease (AD) is the most common cause of dementia and accounts for 60 to 80% of dementia cases [1]. Frontotemporal dementia (FTD) is the second most common cause of dementia [2] and represents a group of brain disorders caused by degeneration of the frontal and/or temporal lobes of the brain including behavior variant frontotemporal dementia (bvFTD), primary progressive aphasias (PPA), and sematic variant primary progressive aphasia (svPPA) [3]. The pathological hallmark of AD includes the deposition of β-amyloid (Aβ) aggregates in the form of senile plaques (SP) and abnormally phosphorylated tau in the form of neurofibrillary tangles (NFT) [4]. A subset of FTD patients, also referred to as with Pick’s disease, have abnormal accumulation of protein inside nerve cells in the damaged areas of the brain. These Pick bodies contain an abnormal form of tau, but the structure and folding of tau filaments are different between Pick’s disease and AD [5].

Both AD and FTD have familial and sporadic forms of the diseases. Amyloid beta precursor protein (*APP*) [6], presenilin 1 (*PSEN1*) [7], and presenilin 2 (*PSEN2*) [8, 9] are AD familial risk genes explaining < 0.5% of AD cases with age of onset typically between 30–50 years of age, while progranulin (*GRN*) [10] and microtubule-associated protein tau (*MAPT*) [11–13] are FTD familial risk genes [2]. Apolipoprotein E (*APOE*) was suggested to play a role in late onset Alzheimer’s disease (LOAD), the sporadic form of AD, long before GWAS era [14]. GWAS confirmed role of variants in *APOE* but additionally identified clusterin (*CLU*), phosphatidylinositol binding clathrin assembly protein (*PICALM*) [15], bridging integrator 1 (*BIN1*) [16], CD2 associated protein (*CD2AP*) [17, 18], complement C3b/C4b receptor 1 (*CR1*) [19], ATP binding cassette subfamily A member 7 (*ABCA7*) [20], and CD33 molecule (*CD33*) [17, 21] to be associated with LOAD. Meta-analysis of 74,046 individuals by International Genomics of Alzheimer’s Project (I-GAP) revealed 19 GWAS significant AD loci including 11 novel loci [21]. These genes were implicated in cholesterol metabolism (*APOE*, *CLU*, and *ABCA7*), immune response (*CR1*, *CD33*, *CLU*, *ABCA7*), and endocytosis (*BIN1*, *PICALM*, *CD2AP*) [22].

Studies have shown that loss-of-function mutations in *GRN*, which encodes a neurotrophic factor, cause familial FTLD, a progressive neurodegenerative disease affecting ∼10% of early-onset dementia patients. Rs646776 was shown to be associated with plasma progranulin levels (*p* = 1.7 x 10^−30^) and replicated in two independent series of 508 controls (*p* = 1.9 x 10^−19^) and 197 FTLD patients (*p* = 6.4 x 10^−12^) [23] with each copy of the minor C allele decreasing progranulin levels by ∼15%. In addition, common variant rs5848 in the 3’ UTR region of the *GRN* gene conferred risk of FTD [24] and LOAD [25], and its genotype was associated with serum progranulin level [26] as well. Rs5848 is also an eQTL variant for *GRN* transcript level in nerve tibial (q-value = 3.19 x 10^−11^) and artery tibial (q-value = 1.66 x 10^−9^) (data source: GTEx V6p version). In addition to the well-known AD and FTD genes, we have previously identified two genome wide significant variants associated with CSF Aβ_42_ levels [27] using Alzheimer's Disease Neuroimaging Initiative (ADNI) samples. In total, 13 variants implicated in AD or FTD, or associated with CSF Aβ_42_ levels were included in the wave 1 of this study for further interrogation.

To elicit additional insight on these 13 SNPs previously implicated in AD and FTD, phenome-wide association studies (PheWAS) were performed using the database from the personal genetics company 23andMe, Inc. to identify phenotypes (both dementia and non-dementia traits) associated with these variants of interest. UK Biobank PheWAS results were also looked up via Open Target Genetics [28]. Since then newer waves of GWAS meta-analysis including samples from UK Biobank using family history as proxy and Alzheimer’s Disease Sequencing Project (ADSP) allows identification of additional novel risk loci [29–32]. In wave 2, we further include 30 variants from the more recent GWAS meta-analyses [29–32] or variants identified earlier but not prioritized in wave 1 PheWAS. PheWAS approach has been previously applied to BioVU, Vanderbilt's DNA biobank where phenotypes are defined by EMR records namely ICD codes [33], or the 23andMe research database where phenotypes are defined by self-reports [34], or both [35]. It has the potential of validating target, nominating treatment indication and/or assessing safety signal especially if the effect of a genetic variant mimics the pharmacotherapy effect [36]. Given that the genes implicated in AD/FTD were implicated in cholesterol metabolism (*APOE*, *CLU*, and *ABCA7*) and immune response (*CR1*, *CD33*, *CLU*, *ABCA7*, *TREM2*, *SPPL2A*, *SCIMP*, *HLA-DRB1*), It is foreseeable that some of the AD variants may be also associated with metabolic/cardiovascular and/or immune-related traits. Recent development of using genetic variants as an instrument variable in GWAS summary statistics based Mendelian randomization (MR) [37] provides another means to dissect the pleiotropy vs. causal relationship between related traits.

## Materials and methods

### Study participants

#### Cohort 1: 23andMe

All individuals included in the analyses were research participants of 23andMe who have provided electronic informed consent, DNA samples for genetic testing, and answered surveys online. The study was conducted according to human subject protocol, which was reviewed and approved by Ethical & Independent Review Services, a private institutional review board (http://www.eandireview.com). It is also consistent with the procedures involving experiments on human subjects in accord with the ethical standards of the Committee on Human Experimentation of the institution in which the experiments were done or in accord with the Helsinki Declaration of 1975. All data was completely anonymized and de-identified before access by the analyst for data analysis.

As described previously [38], DNA samples have been genotyped on one of four genotyping platforms. The v1 and v2 platforms were variants of the Illumina HumanHap550+ BeadChip (Illumina, San Diego, CA, USA), including about 25 000 custom single-nucleotide polymorphisms (SNPs) selected by 23andMe, with a total of about 560 000 SNPs. The v3 platform was based on the Illumina OmniExpress+ BeadChip, with custom content to improve the overlap with the v2 array, with a total of about 950 000 SNPs. The v4 platform is a fully custom array, including a lower redundancy subset of v2 and v3 SNPs with additional coverage of lower-frequency-coding variation, and about 570 000 SNPs. S1 Table shows which 23andMe genotype platform (v1-v4) the tested variant is genotyped on. It also shows the imputation statistics for the tested variant, including the average imputation dosages for the first (A) and second (B) alleles (freq.a and freq.b) and the average and minimum imputation quality across all batches (avg.rsqr and min.rsqr). The r^2^ statistic is used to measure imputation quality, which range from 0 (worst) to 1 (best). The batch effect test is an F test from an ANOVA of the SNP dosages against a factor representing imputation batch.

Only participants enrolled by 2015 were included in this analysis. A similar approach using the same research database was previously described [34]. We tested the association with more than 1000 well-curated phenotypes (S2 Table), which were distributed among different phenotypic categories (e.g. cognitive, autoimmune, psychiatric etc.). GWAS were previously performed on these well-curated phenotypes and confirmed to replicate known associations and not to generate spurious false positives. For our standard PheWAS, we restricted participants to a set of individuals who have > 97% European ancestry, as determined through an analysis of local ancestry [39]. Briefly, this algorithm first partitioned phased genomic data into short windows of about 100 SNPs. Within each window, we used a support vector machine (SVM) to classify individual haplotypes into one of 31 reference populations. The SVM classifications were then fed into a hidden Markov model (HMM) accounting for switch errors and incorrect assignments as well as generating probabilities for each reference population in each window. Finally, we used simulated admixed individuals to recalibrate the HMM probabilities so that the reported assignments were consistent with the simulated admixture proportions. The reference population data is derived from public datasets (the Human Genome Diversity Project, HapMap, and 1000 Genomes–participants provided informed consent and all data was completely anonymized and de-identified before access and analysis), as well as 23andMe customers who have reported having four grandparents from the same country. A maximal set of unrelated individuals was chosen for each phenotype using a segmental identity-by-descent (IBD) estimation algorithm [40]. Individuals were defined as related if they shared more than 700 cM IBD, including regions where the two individuals shared either one or both genomic segments identical-by-descent. This level of relatedness (roughly 20% of the genome) corresponded approximately to the minimal expected sharing between first cousins in an outbred population.

The imputed dosages rather than best-guess genotypes were used for association testing in PheWAS. Participant genotype data were imputed against the September 2013 release of 1000 Genomes Phase1 reference haplotypes, phased with ShapeIt2 [41]. Genotype data for research participants were generated from four versions of genotyping chips as described previously [38]. We phased and imputed data for each genotyping platform separately. We phased using a phasing tool Finch, which implements the Beagle haplotype graph-based phasing algorithm [42], modified to separate the haplotype graph construction and phasing steps. Finch extended the Beagle model to accommodate genotyping error and recombination, to handle cases where there were no consistent paths through the haplotype graph for the individual being phased. We constructed haplotype graphs for European and non-European samples on each 23andMe genotyping platform from a representative sample of genotyped individuals, and then performed out-of-sample phasing of all genotyped individuals against the appropriate graph.

In preparation for imputation, we split phased chromosomes into segments of no more than 10,000 genotyped SNPs, with overlaps of 200 SNPs. We excluded SNPs with Hardy-Weinberg equilibrium *p* < 10^−20^, call rate < 95%, or with large allele frequency discrepancies compared to European 1000 Genomes reference data. Frequency discrepancies were identified by computing a 2x2 table of allele counts for European 1000 Genomes samples and 2000 randomly sampled 23andMe customers with European ancestry and identifying SNPs with a chi squared *p* < 10^−15^. We imputed each phased segment against all-ethnicity 1000 Genomes haplotypes (excluding monomorphic and singleton sites) using Minimac2 [43], using 5 rounds and 200 states for parameter estimation.

Association test results were computed using logistic regression for case control comparisons, or linear regression for quantitative traits. For survival traits, association test results using Cox proportional hazards regression were computed. We assumed additive allelic effects and included covariates for age, gender, and the top five principal components to account for residual population structure. The association test *p* value reported was computed using a likelihood ratio test, which was shown to be a better choice despite of its computational demands [44]. We reported raw p-values for the PheWAS association results, but interpret the results taking into account the number of variants and traits tested. An association with *p* < 0.05 / (13*1,234) = 3.12x10^-6^ was deemed to be significant association, other associations with FDR < 0.05 was deemed to be suggestive associations.

#### Cohort 2: UK Biobank (UKB)

Pre-computed UK Biobank PheWAS results based on Neale lab UK Biobank summary statistics were looked up via Open Target Genetics [28] (genetics.opentargets.org) for side by side comparison with PheWAS results based on the 23andMe cohort. There were three version of UKB results accessed via Open Target Genetics, Neale v1 PheWAS results were accessed in November 2019, and Neale v2 PheWAS results (http://www.nealelab.is/uk-biobank) were accessed in July 2020. UKB SAIGE is yet a different version of UKB PheWAS results by University of Michigan (http://pheweb.sph.umich.edu/SAIGE-UKB/about). There are 4593 traits in total for the Neale v2 PheWAS analysis. We report raw p-values for the PheWAS association results, but interpret the results taking into account the number of variants and traits tested. An association with *p* < 0.05 / (48*4593) = 2.27 x 10^−7^ was deemed to be significant association. No additional adjustment was made for Neale v1 PheWAS results (a few traits present in v1 were not present in v2) and/or UKB SAIGE PheWAS.

### Whole-genome genetic correlations between significant PheWAS traits

For convenience of collecting whole-genome summary statistics, AD summary statistics from Jansen et al study [30] was used to calculate genetic correlation with other traits using LD Hub (v1.9.3) [45], which is a centralized database of summary-level GWAS results and a web interface for LD score regression (LDSC) [46].

### Directional horizontal pleiotropy vs causal relationship

For the multiple clinical phenotypes associated with the AD/FTD variants identified in the PheWAS analysis, we attempted to untangle the relationship between trait A and trait B to determine if genetic variants impact trait A (also called exposure in the literature) and trait B (also called outcome in the literature) independently, or genetic variants’ effect on trait B is mediated by trait A (or vice versa). We applied MR Egger intercept test [47, 48] to test directional horizontal pleiotropy, where the variants affect both trait A (e.g. CAD) and trait B (e.g. AD) independently. MR uses genetic variants as a proxy for an environmental exposure/trait A (e.g. CAD), assuming that: 1) the genetic variants are associated with the exposure/trait A; 2) the genetic variants are independent of confounders in the exposure-outcome association; 3) the genetic variants are associated with the outcome only via their effect on the exposure, i.e. there is no horizontal pleiotropy whereby genetic variants have an effect on an outcome (e.g. AD) independent of its influence on the exposure (e.g. CAD). If the MR Egger intercept test had a significant p-value (*p* < 0.05) (i.e. violating assumption #3 from the MR analysis), the pair of traits was excluded from the bi-directional, two-sample MR test using inverse variance weighted (IVW) method among traits identified in the PheWAS study. In this case (Egger intercept *p* < 0.05), the gene-outcome vs gene-exposure regression coefficient is estimated using MR Egger regression to correct for the bias due to directional pleiotropy, under a weaker set of assumptions than typically used in MR [49]. Both IVW and MR Egger regression however do not protect against violation of assumption #2. The MR analysis is also only feasible if there is sufficient information from MR Base [50] for analysis or if the information could be supplemented by manually adding GWAS results from publications, e.g. the recent AD meta-analysis by Jansen et al. [30] In the MR analysis, we primarily leveraged variants implicated in a trait from public summary statistics (pre-compiled as a set of instruments from NHGRI-EBI GWAS Catalog [51] in the MRInstruments R package v0.3.2 https://github.com/MRCIEU/MRInstruments) as an individual variant is unlikely to be powerful enough as an instrument variable unless the effect size is large. Instrument variables were constructed using the default independent genome wide significant SNPs (*p* < = 5 x 10^−8^) for AD and other diseases/risk factors except for FTD where a p-value threshold of *p* < = 6 x 10^−6^ was used because of the smaller GWAS samples size [52]. We assessed if bi-directional causal relationships exist between AD and a number of significant PheWAS traits identified in the PheWAS analyses. For FTD, only directional MR analysis was performed using FTD as the exposure as only top GWAS hits were available publicly. All analyses were performed using the MR-Base ‘TwoSampleMR’ v0.5.4 package [50] in R and MR test with nominal *p* < 0.05 using inverse variance weighted and/or MR Egger method was reported. A *p* < 0.05/ # of PheWAS traits examined is considered significant, while a *p* < 0.05 is considered suggestive.

## Results

Thirteen variants were successfully imputed from the four genotyping platforms in the 23andMe cohort with the average and minimum imputation quality across all batches (avg.rsqr and min.rsqr) ranging from 0.96 to 1 for avg.rsqr (average across 13 variants was 0.995) and 0.86 to 1 for min.rsqr (average = 0.982) (S1 Table).

### AD-risk variants are highly associated with neurological, longevity, metabolic, cardiovascular, eye, and immune-related traits

**Fig 1:**
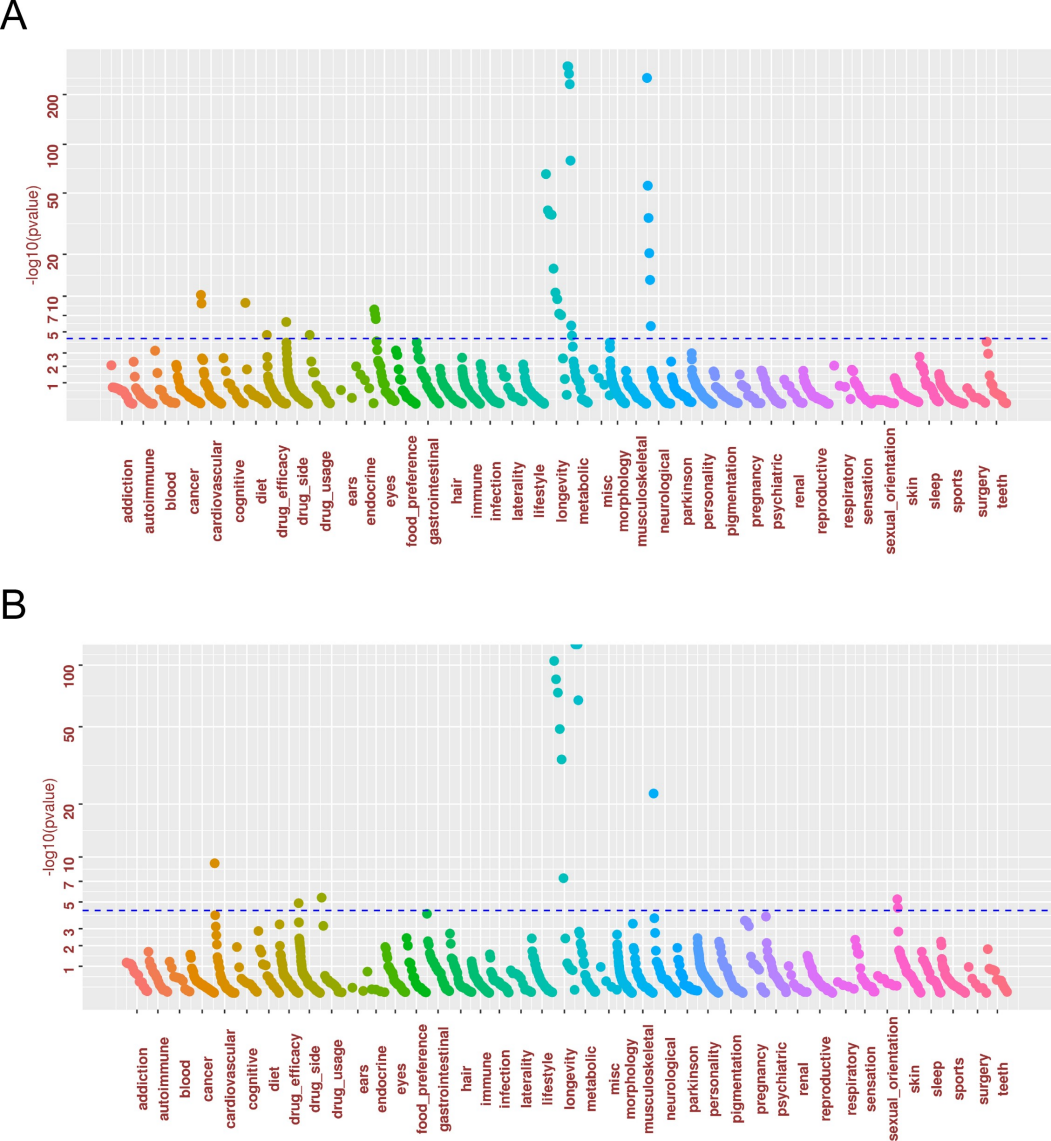
PheWAS association plot for *APOE* variants. A grey line is drawn at Positions *p* = 5 x 10^−5^ (a score of about 4.3), which is a threshold for significance after controlling for the Family-Wise-Error-Rate (FWER) using Bonferroni correction. (a) rs429358; (b) rs7412.

**Fig 2:**
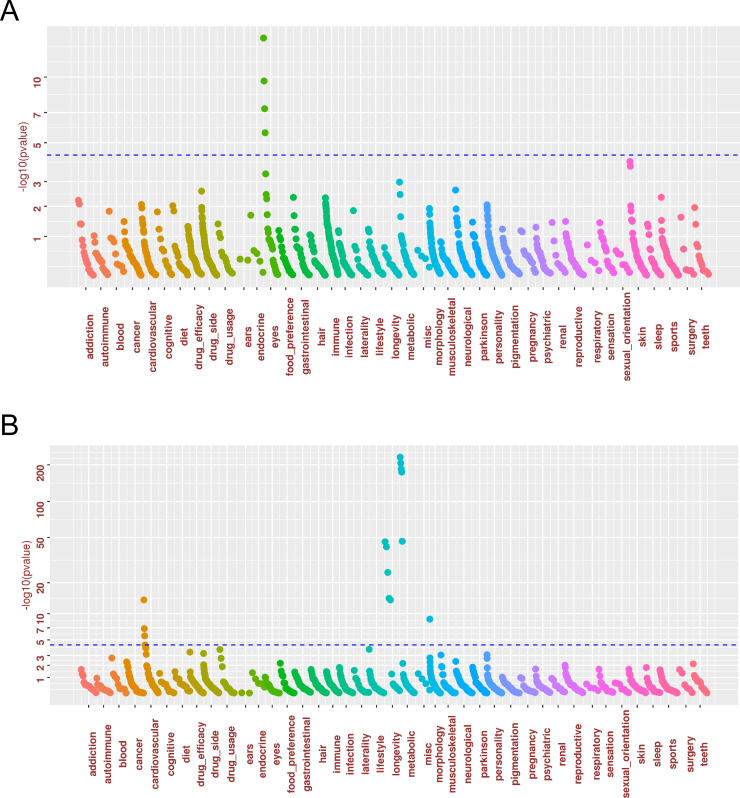
PheWAS association plot. A grey line is drawn at Positions *p* = 5 x 10^−5^ (a score of about 4.3), which is a threshold for significance after controlling for the FWER using Bonferroni correction. (a) rs11136000 (*CLU*); (b) rs646776.

Selected PheWAS findings were summarized in Tables 1 and 2 for wave 1 and wave 2 PheWAS alongside the known associations reported in the NHGRI-EBI GWAS Catalog [51] for the SNPs previously associated with LOAD and/or FTD. The list of PheWAS association results using the 23andMe cohort with FDR < 0.05 is available as S3 Table, while the full list of PheWAS association results is available from S4 Table. An association in the 23andMe cohort with *p* < 0.05 / (13*1,234) = 3.12 x 10^−6^ was deemed to be significant association, other associations with FDR < 0.05 was deemed to be suggestive associations. A number of the known associations was replicated. In addition, novel associations were identified. The two SNPs, rs429358 and rs7412, defining APOE ε2/ε3/ε4 alleles were known to be associated with multiple neurological, longevity, metabolic and cardiovascular traits (Fig 1, Table 1, and S3 Table). Subjects carrying the minor T allele of rs7412 are *APOE* ε2 protective allele carriers and subjects carrying the minor C allele of rs429358 are *APOE* ε4 risk allele carriers. PheWAS identified significant associations with metabolic traits (high cholesterol or taking drugs to lower cholesterol, body mass index (BMI)), neurological traits (AD family history, AD, cognitive decline, mild cognitive impairment, memory problems), longevity traits (nonagenarian—at least 90 years old, healthy old—over age 60 with no cancer or disease, centenarian family), cardiovascular diseases (coronary artery disease (CAD), metabolic and heart disease), and eye problems (nearsightedness, glasses usage, myopia vs. hyperopia), and serious side effects from statins (rs429358, *p* = 7.14 x 10^−7^). The directionality of association is consistent with the protective vs. risk effect of two *APOE* SNPs in that the minor allele of rs7412 was associated with lower risk of high cholesterol, while the minor allele of rs429358 was associated with higher risk of high cholesterol (*p* = 6.6 x 10^−295^). Additional suggestive associations were identified (FDR < 0.05) for age-related macular degeneration (AMD) or blindness (rs429358 *p* = 9.59 x 10^−5^, FDR = 0.004). Interestingly, rs11136000 from *CLU* is also strongly associated with multiple eye phenotypes (nearsightedness, myopia, glasses, astigmatism) (Table 1, Fig 2, and S3 Table). Subjects carrying the minor allele of rs429358 had lower chance of nearsightedness (*p* = 1.4 x 10^−8^), while subjects carrying the minor allele of rs11136000 had higher chance of nearsightedness (*p* = 4.5 x 10^−15^). For the overlapping phenotypes, UK Biobank PheWAS results largely supported the 23andMe PheWAS findings.

**Table 1:** Wave 1 PheWAS results. P-values are in bold font if passing the PheWAS multiple testing correction threshold of 3.12x10^-6^ in 23andMe cohort or 2.32x10^-7^ in UKB cohort.

SNP	Chr	BP*	Freq_B_**	Alleles**	Gene	Published associated with AD		Selected known associations*** from Open Targets Genetics		Phenotype Group	Selected 23andMe PheWAS Results						Selected UKBB PheWAS results					
						Reported p-value	Analysis	DISEASE/TRAIT	PubMed ID		DISEASE/TRAIT	β**	*OR*	*p*	*N Cases*	*N Overall*	DISEASE/TRAIT	β**	*OR*	*p*	*N Cases*	*N Overall*
***Variants associated with FTD***			** **	** **	** **	** **	** **	** **	** **	** **	** **	** **	** **	*** ***	*** ***	*** ***	** **	** **	** **	*** ***	*** ***	*** ***
rs646776	1	109275908	0.78	C/T	*CELSR2—PSRC1*		* *	Progranulin levels	21087763	Neurological												
					* *	* *	* *			Neurological							Alzheimer's disease/dementia | illnesses of father	-0.038	0.963	6.47E-03	15022	312666
					* *	* *	* *			longevity	Over age 60 with no cancer or disease	-0.10	0.91	**1.81E-14**	20409	234228						
					* *	* *	* *	Total cholesterol	19060911, 25961943, 29403010	metabolic	high_cholesterol	0.26	1.29	**2.74E-207**	112221	219875	High cholesterol | non-cancer illness code, self-reported	0.174	1.191	**4.27E-95**	43957	361141
					* *	* *	* *	LDL cholesterol	19060911, 19060910, 18193044, 25961943	metabolic				** **								
					* *	* *	* *			metabolic	iqb.low_hdl	0.15	1.17	**3.37E-47**	36339	154349						
					* *	* *	* *	Coronary artery disease	21239051, 21378988	cardiovascular	Coronary artery disease	0.12	1.13	**1.79E-14**	13648	329205	Angina | non-cancer illness code, self-reported	0.125	1.133	**6.39E-15**	11370	361141
					* *	* *	* *	Myocardial infarction (early onset)	19198609	cardiovascular	Heart attack	0.11	1.12	**1.37E-07**	7573	334019	Heart attack/myocardial infarction | non-cancer illness code, self-reported	0.118	1.126	**2.49E-10**	8239	361141
					* *	* *	* *	Response to statin therapy	20339536	pharmacogenomics				** **						** **		
					* *	* *	* *			anthropomorphic	Height	-0.05		**1.80E-09**		355080	Standing height	-0.095		**1.21E-07**		360388
					* *	* *	* *			eyes	Wear cosmetic contact lenses	-0.22	0.80	1.88E-04	788	37145	Which eye(s) affected by myopia (short sight): Right eye^a^	-0.039	0.961	3.87E-01	1462	26943
rs5848	17	44352876	0.30	C/T	*GRN*	* *	* *	Progranulin levels	29186428	Neurological												
					* *	* *	* *			Neurological	Parkinson’s disease	0.07		1.38E-05	10082	396801	Parkinsons disease | non-cancer illness code, self-reported	0.165	1.179	8.63E-03	652	361141
					* *	* *	* *			Neurological							Diagnoses—main ICD10: G20 Parkinson's disease^a^	0.219		1.78E-01	97	337199
					* *	* *	* *			Neurological							Illnesses of father: Parkinson's disease^a^	0.051		5.78E-03	7541	292053
					* *	* *	* *			Neurological							Illnesses of mother: Parkinson's disease^a^	-0.022		3.30E-01	5094	308780
					* *	* *	* *			Neurological							Illnesses of siblings: Parkinson's disease^a^	-0.001		9.77E-01	1345	259921
					* *	* *	* *			Neurological							Alzheimer's disease/dementia | illnesses of mother	0.026	1.026	7.66E-03	28507	331041
					* *	* *	* *			Physical							Impedance of leg (right)	-0.39		6.58E-06		354817
					* *	* *	* *	Mean platelet volume	27863252	Autoimmune disease biomarker							Mean platelet (thrombocyte) volume	0.048		2.76E-61		350470
					* *	* *	* *	Platelet distribution width									Platelet distribution width	0.017		2.82E-35		350470
***Variants associated with Alzheimer's disease***					*** ***	*** ***	*** ***	Platelet count	*** ***	*** ***	*** ***	*** ***	*** ***	*** ***	*** ***	*** ***	Platelet count	-1.861		1.75E-32		350474
rs3818361	1	207611623	0.81	A/G	*CR1*	5.40E-14	IGAP stage 1	Alzheimer's disease (late onset)	24162737	Neurological							Alzheimer's disease/dementia | illnesses of mother	-0.05	0.951	5.55E-06	28507	331041
					* *	* *	* *	Family history of Alzheimer's disease	29777097	Neurological												
rs744373	2	127137039	0.29	A/G	*BIN1*	2.12E-16	IGAP stage 1	Alzheimer's disease (late onset)	24162737; 21390209; 21627779	Neurological	alzheimers	0.09		1.60E-01	645	157843						
								Family history of Alzheimer's disease	29777097	Neurological	iqb.alzheimers_fh	0.04		1.76E-04	25610	87378	Alzheimer's disease/dementia | illnesses of mother	0.06	1.062	**1.93E-10**	28507	331041
rs9349407	6	47485642	0.74	C/G	*CD2AP*	3.92E-07	IGAP stage 1	Alzheimer's disease (late onset)	24162737; 21460841; 21460840	Neurological	iqb.alzheimers_fh	-0.02		3.79E-02	25610	87378						
					* *			Mean platelet volume	27863252	Autoimmune disease biomarker							Mean platelet (thrombocyte) volume	0.037		**1.89E-37**		350470
					* *			Platelet distribution width	27863252	Hematological measurement							Platelet distribution width	0.017		**7.00E-37**		350470
					* *			Platelet count	27863252	Hematological measurement							Platelet count	-1.352		**2.27E-18**		350474
					* *			High light scatter reticulocyte percentage of red cells	27863252	Hematological measurement							High light scatter reticulocyte percentage	-0.006		**1.50E-10**		344729
					* *	* *	* *			Anthropomorphic	height	-0.03		5.53E-06		355080	Standing height	-0.131		**3.50E-15**		360388
rs11136000	8	27607002	0.40	C/T	*CLU*	1.72E-16	IGAP stage 1	Alzheimer's disease (late onset)	24162737; 19734902	Neurological												
					* *			Family history of Alzheimer's disease	29777097	Neurological	iqb.alzheimers_fh	-0.03		2.31E-03	25610	87378	Alzheimer's disease/dementia | illnesses of mother	-0.045	0.956	3.25E-07	28507	331041
					* *	* *	* *			eyes	nearsightedness	0.05		**4.51E-15**	123722	102325	Which eye(s) affected by myopia (short sight): Left eye^a^	-0.065		1.02E-01	1377	26943
					* *	* *	* *			eyes	Astigmatism	0.03		**2.45E-06**	77240	80566	Which eye(s) affected by astigmatism: Left eye^a^	0.058		1.66E-01	1450	8863
rs116953792	10	93703269	0.03	G/T	*FRA10AC1*	* *	* *	Cerebrospinal fluid Aβ1–42 levels	26252872	Neurological	-			-			Illnesses of siblings: Alzheimer's disease/dementia	0.201		4.43E-02	1468	259921
rs3851179	11	86157598	0.37	C/T	*PICALM*	2.84E-15	IGAP stage 1	Alzheimer's disease (late onset)	24162737; 19734902	Neurological												
					* *	* *	* *	Alzheimer's disease in APOE ε4- carriers	25778476	Neurological												
					* *	* *	* *	Family history of Alzheimer's disease	29777097	Neurological	iqb.alzheimers_fh	-0.03		1.18E-02	25610	87378	Alzheimer's disease/dementia | illnesses of mother	-0.039	0.962	1.32E-05	28507	331041
					* *	* *	* *			Neurological							Alzheimer's disease/dementia | illnesses of father	-0.048	0.954	6.69E-05	15022	312666
					* *	* *	* *			Neurological	migraine	-0.03		4.40E-05	74955	353214						
rs1503351	15	96814290	0.05	A/G	*SPATA8—RN7SKP181*		* *	Cerebrospinal fluid Aβ1–42 levels	26252872	Neurological							Alzheimer's disease/dementia | illnesses of mother	0.046	1.047	3.59E-02	28507	331041
rs3764650	19	1046521	0.91	G/T	*ABCA7*	3.22E-07	IGAP stage 1	Alzheimer's disease (late onset)	24162737; 21460840	Neurological							Alzheimer's disease/dementia | illnesses of mother	-0.043	0.958	3.81E-03	28507	331041
					* *	* *	* *			Treatment/medication							Mean sphered cell volume	-0.127		**7.01E-09**		344729
rs429358	19	44908684	0.86	C/T	*APOE*	* *	* *	Brain imaging	20100581, 29860282	Neurological												
					* *	* *	* *	Alzheimer's disease (late onset)	24162737	Neurological	alzheimers	-1.12		**2.39E-56**	645	157843	Alzheimer's disease^b^	-1.9	0.15	**2.34E-58**	404	402787
					* *	* *	* *										Delirium dementia and amnestic and other cognitive disorders^b^	-0.79	0.454	**3.86E-60**	1970	404353
					* *	* *	* *	Cognitive decline	28078323	Neurological	cognitive_decline	-0.42		**1.84E-35**	3584	96928	Non-cancer illness code, self-reported: dementia/alzheimers/cognitive impairment^a^	-1.600		**7.34E-14**	83	337159
					* *	* *	* *	Alzheimer's disease progression score	29860282	Neurological				** **						** **		
					* *	* *	* *	Family history of Alzheimer's disease	29777097	Neurological	iqb.alzheimers_fh	-0.50		**3.16E-252**	25610	87378	Alzheimer's disease/dementia | illnesses of father	-0.529	0.589	**1.53E-243**	15022	312666
					* *	* *	* *			Neurological				** **			Alzheimer's disease/dementia | illnesses of siblings	-0.591	0.554	**3.36E-36**	1609	279062
					* *	* *	* *			Neurological				** **			Alzheimer's disease/dementia | illnesses of mother	-0.597	0.55	**0.00E+00**	28507	331041
					* *	* *	* *	Cerebral amyloid deposition	26252872, 29860282	Neurological	iqb.mild_cognitive_imp_fh	-0.27		**4.39E-21**	5497	54813						
					* *	* *	* *	Cerebrospinal fluid Aβ1–42 levels	25027320	Neurological												
					* *	* *	* *	Lewy body disease	25188341, 29263008	Neurological												
					* *	* *	* *	age-related macular degeneration	26691988	eyes												
					* *	* *	* *			eyes	Nearsightedness	0.05		**1.45E-08**	123722	226047	Reason for glasses/contact lenses: For short-sightedness, i.e. only or mainly for distance viewing such as driving, cinema etc (called 'myopia')^a^	0.032	1.033	9.25E-03	26943	335700
					* *	* *	* *	HDL cholesterol	25961943, 29403010	metabolic	Body mass index	0.09		**1.95E-06**		344351	Body mass index (BMI)	0.112		**3.54E-13**		359983
					* *	* *	* *	LDL cholesterol	29403010	metabolic	high_cholesterol	-0.36		**6.64E-295**	112221	219875	High cholesterol | non-cancer illness code, self-reported	-0.261	0.77	**3.66E-160**	43957	361141
					* *	* *	* *	Total cholesterol levels	29403010	metabolic	iqb.low_hdl	-0.23		**3.27E-80**	36339	154349				** **		
					* *	* *	* *			metabolic/family illness				** **			Diabetes | illnesses of mother	0.093	1.098	**4.96E-16**	30772	331142
					* *	* *	* *			cardiovascular	Coronary artery disease	-0.12		**5.93E-11**	13648	329205	Cholesterol lowering medication | medication for cholesterol, blood pressure, diabetes, or take exogenous hormones	-0.248	0.781	**1.56E-81**	24247	193148
					* *	* *	* *			cardiovascular	Heart attack	-0.15		**1.71E-09**	7573	334019	Ischemic heart disease^b^	-0.101	0.904	**3.89E-16**	31355	408458
					* *	* *	* *			Physical				** **			Leg fat percentage (left)	0.149		**1.79E-18**		354791
					* *	* *	* *	physical activity	29899525	life style				** **								
					* *	* *	* *	Parental lifespan	29030599	longevity	At least 90 years old	0.37		**3.80E-37**	5964	417602						
					* *	* *	* *	Platelet count	27863252	lab measurement												
					* *	* *	* *	Red cell distribution width	27863252	lab measurement												
					* *	* *	* *	C-reactive protein levels	29403010	protein biomarker												
rs7412	19	44908822	0.08	C/T	*APOE*	* *	* *	Alzheimer's disease	28714976	Neurological	alzheimers	-0.43		1.91E-04	645	157843						
					* *	* *	* *	Family history of Alzheimer's disease	29777097	neurological/family illnesses	iqb.alzheimers_fh	-0.20		**1.66E-23**	25610	87378	Alzheimer's disease/dementia | illnesses of mother	-0.217	0.805	**2.45E-43**	28507	331041
					* *	* *	* *	Mortality	27029810	longevity	At least 90 years old	0.18		**4.54E-08**	5964	417602				** **		
					* *	* *	* *	Response to statins (LDL cholesterol change)	22331829	pharmacogenomics				** **						** **		
					* *	* *	* *	LDL cholesterol	23067351, 28371326, 28548082, 28334899	metabolic				** **						** **		
					* *	* *	* *	HDL cholesterol	28270201	metabolic	iqb.low_hdl	-0.29		**3.24E-68**	36339	154349				** **		
					* *	* *	* *	Cholesterol, total	25961943, 28548082, 28270201, 28334899	metabolic	high_cholesterol	-0.62		**0.00E+00**	112221	219875	High cholesterol | non-cancer illness code, self-reported	-0.346	0.707	**2.04E-158**	43957	361141
					* *	* *	* *			metabolic	Presence of any metabolic or heart-related disease	-0.23		**2.06E-105**	166416	318382	Heart attack/myocardial infarction | non-cancer illness code, self-reported	-0.173	0.841	**1.54E-09**	8239	361141
					* *	* *	* *			metabolic										** **		
					* *	* *	* *			Physical							Trunk fat mass	0.156		**3.88E-12**		354597
					* *	* *	* *			Physical							Whole body fat mass	0.267		**2.97E-11**		354244
					* *	* *	* *			Physical							Hip circumference	0.259		**5.83E-11**		360521
					* *	* *	* *										Cholesterol lowering medication | medication for cholesterol, blood pressure, diabetes, or take exogenous hormones	-0.351	0.704	**5.26E-92**	24247	193148
					* *	* *	* *	Lipoprotein (a) levels	28512139	lab measurement										** **		
					* *	* *	* *	Lipoprotein-associated phospholipase A2 activity change in response to darapladib	28753643	pharmacogenomics										** **		
					* *	* *	* *	Lipoprotein phospholipase A2 activity	28753643											** **		
					* *	* *	* *	Coronary artery disease	29212778, 28714975	cardiovascular	Coronary artery disease	-0.16		**7.13E-10**	13648	329205	Angina | vascular/heart problems diagnosed by doctor	-0.19	0.827	**7.52E-15**	11372	360420
					* *	* *	* *	Pulse pressure	28135244	vital sign												
rs3865444	19	51224706	0.69	A/C	*CD33*	5.12E-08	IGAP stage 1	Alzheimer's disease	21460841; 24162737; 28714976; 21460840	Neurological	-			-			Non-cancer illness code, self-reported: dementia/alzheimers/cognitive impairment^a^	0.037		8.22E-01	83	337159
					* *					Neurological							Alzheimer's disease^b^	0.236	1.266	1.65E-03	404	402787
					* *	* *	* *	Family history of Alzheimer's disease	29777097	Neurological							Alzheimer's disease/dementia | illnesses of mother	0.022	1.023	1.46E-02	28507	331041
								Platelet count	27863252	Hematological measurement							Platelet count	1.310		**7.15E-19**		350474
								White blood cell count	27863252	Hematological measurement							White blood cell (leukocyte) count	0.042		**2.35E-16**		350470
								Plateletcrit	27863252	Hematological measurement							Plateletcrit	0.001		**9.43E-22**		350471
								Lymphocyte counts	27863252	Hematological measurement										** **		
										Hematological measurement							Neutrophill count	0.024		**2.74E-11**		349856
										Hematological measurement							Eosinophill count	0.012		**3.99E-10**		349856
																	Monocyte count	0.003		**5.00E-08**		349856

iqb.alzheimers_fh: cases report having any of their grandparents, parents, brothers, sisters, aunts, or uncles ever been diagnosed with AD; Alzheimer: AD, age 55 or older; cognitive_decline: Any report of cognitive impairment or memory loss, age 65 and older, excluding AD cases; iqb.mild_cognitive_imp_fh: "Have any of your grandparents, parents, brothers, sisters, aunts, or uncles ever been diagnosed with mild cognitive impairment (MCI)?"; iqb.low_hdl: Ever told by a medical professional that your high-density lipoprotein is too low; high_cholesterol: High cholesterol or taking drugs to lower cholesterol.

*chromosomal position based on genome build GRCh38 coordinate.

**The Alleles column describes the two possible alleles at the variant location, listed in alphabetical order. In this study, the first allele will be called "A allele" and the second allele will be called the "B allele"; effect (β): The effect size, ln(Odds Ratio [OR]) for binary traits, defined per copy of the B allele.

*** Highlighted associations do not necessarily pass genome wide significance threshold in all PubMed ID citations.

^a^Accessed November 2019

^b^UKB SAIGE, while the rest shall be UKB Neale v2.

**Table 2:** Wave 2 PheWAS results.

SNP	Chr	BP*	Freq_B_**	Alleles**	Gene	Published associated with AD		Selected known association*** from NHGRI-EBI Catalog		Phenotype Group	Selected UKBB PheWAS results					
						Reported p-value	Analysis	DISEASE/TRAIT	PubMed ID		DISEASE/TRAIT	β**	*OR*	*p*	*N Cases*	*N Overall*
***Additional variants associated with Alzheimer's disease***					*** ***	*** ***	*** ***	*** ***	*** ***	*** ***	*** ***	*** ***	*** ***	*** ***	*** ***	*** ***
rs4575098	1	161185602	0.76	A/G	*ADAMTS4*	2.05E-10	Overall (phase 3) (Jansen et al, 2019)	Family history of Alzheimer's disease	30617256; 29777097	Neurological	Alzheimer's disease/dementia | illnesses of mother	-0.060	0.942	**2.84E-09**	28507	331041
					* *	* *	* *	Monocyte percentage of white cells	27863252	Hematological measurement	Monocyte percentage	-0.048		**1.37E-10**		349861
					* *	* *	* *	Platelet distribution width	27863252	Hematological measurement	Platelet distribution width	0.012		**9.62E-16**		350470
					* *	* *	* *			Hematological measurement	Neutrophill count	0.022		**3.85E-08**		349856
					* *	* *	* *	Mean platelet volume	27863252	Autoimmune disease biomarker	Mean platelet (thrombocyte) volume	0.030		**6.06E-23**		350470
					* *	* *	* *	Granulocyte percentage of myeloid white cells	27863252	Hematological measurement						
rs10933431	2	233117202	0.234	C/G	*INPPD5*	8.92E-10	Overall (phase 3) (Jansen et al, 2019)	Alzheimer's disease (late onset)	30617256; 24162737	Neurological	Alzheimer's disease/dementia | illnesses of mother	-0.043	0.958	3.97E-05	28507	331041
rs184384746	3	57192122	0.002	C/T	*HESX1*	*1*.*24E-08*	*AD-by-proxy (phase 2) (Jansen et al*, *2019)*	AD-by-proxy	30617256; 30617256	Neurological	Alzheimer's disease/dementia | illnesses of mother	0.254	1.289	3.21E-02	28507	331041
rs6448453	4	11024404	0.747	A/G	*CLNK*	1.93E-09	Overall (phase 3) (Jansen et al, 2019)	Family history of Alzheimer's disease	30617256; 29777097	Neurological	Alzheimer's disease/dementia | illnesses of mother	-0.049	0.952	4.68E-07	28507	331041
rs7657553	4	11721611	0.71	A/G	*HS3ST1*	*2*.*16E-08*	*Case-control status (phase 1) (Jansen et al*, *2019)*	Alzheimer's disease (late onset)	30617256; 24162737	Neurological						
rs187370608	6	40974457	0.99799	A/G	*TREM2*	1.45E-16	Overall (phase 3) (Jansen et al, 2019)	AD-by-proxy	30617256; 30617256	Neurological	Alzheimer's disease/dementia | illnesses of mother	-0.475	0.622	**8.07E-09**	28507	331041
rs6931277	6	32615580	0.16	A/T	*HLA-DRB1*	*8*.*41E-11*	*Overall (phase 3) (Jansen et al*, *2019)*	Family history of Alzheimer's disease	30617256; 29777097	Immune system						
					* *	* *	* *	Ulcerative colitis	28067908	Immune system	Ulcerative colitis^b^	-0.206	0.8138331	**2.23E-10**	3195	337978
					* *	* *	* *			Immune system	Rheumatoid arthritis | non-cancer illness code, self-reported	0.691	1.997	**2.30E-130**	4017	361141
					* *	* *	* *			Immune system	Celiac disease^b^	-0.702	0.496	**5.99E-54**	1855	336638
					* *	* *	* *			Respiratory system	Asthma | non-cancer illness code, self-reported	0.162	1.176	**8.57E-68**	41934	361141
					* *	* *	* *			Neurological	Multiple sclerosis | non-cancer illness code, self-reported	-0.366	0.693	**1.22E-13**	1326	361141
					* *	* *	* *	Inflammatory bowel disease	28067908	Immune system	Inflammatory bowel disease and other gastroenteritis and colitis	-0.140	0.869	2.87E-07	4528	339311
					* *	* *	* *			Endocrine system	Type 1 diabetes	0.617	1.853	**1.55E-60**	2660	391416
					* *	* *	* *	Type 2 diabetes	29358691	Endocrine system	Type 2 diabetes^b^	0.119	1.126	**4.63E-17**	18945	407701
					* *	* *	* *	White blood cell count	27863252	Hematological measurement	White blood cell (leukocyte) count	0.099		**1.29E-58**		350470
					* *	* *	* *	Neutrophil count	27863252	Hematological measurement	Neutrophill count	0.074		**4.43E-67**		349856
					* *	* *	* *	Monocyte percentage of white cells	27863252	Hematological measurement	Monocyte percentage	-0.044		**3.49E-08**		349861
rs1859788	7	100374211	0.697	A/G	*ZCWPW1*	2.22E-15	Overall (phase 3) (Jansen et al, 2019)	Family history of Alzheimer's disease	30617256; 29777097	Neurological	Alzheimer's disease/dementia | illnesses of mother	0.044	1.045	1.80E-06	28507	331041
					* *					Hematological measurement	Haemoglobin concentration	0.016		**4.94E-10**		350474
					* *						Pulse rate, automated reading	-0.169		**6.58E-09**		340162
rs7810606	7	143411065	0.50	C/T	*EPHA1*	*3*.*59E-11*	*Overall (phase 3) (Jansen et al*, *2019)*	Alzheimer's disease (late onset)	30617256; 24162737; 21460840	Neurological	Alzheimer's disease/dementia | illnesses of father	-0.042322	0.9585607	2.63E-04	15022	312666
rs114360492	7	146252937	0.000259	C/T	*CNTNAP2*	2.10E-09	Overall (phase 3) (Jansen et al, 2019)		30617256							
rs11257238	10	11675398	0.62	C/T	*ECHDC3*	*1*.*26E-08*	*Overall (phase 3) (Jansen et al*, *2019)*	Alzheimer's disease (late onset)	30617256; 24162737; 28092683	Neurological						
					* *	* *	* *			Immune system	Ulcerative colitis	0.0582689	1.06	1.52E-03	6687	26405
rs2081545	11	60190907	0.617	A/C	*MS4A6A*	1.55E-15	Overall (phase 3) (Jansen et al, 2019)	Alzheimer's disease (late onset)	30617256; 24162737; 21460840	Neurological	Alzheimer's disease/dementia | illnesses of mother	0.028	1.028	1.59E-03	28507	331041
					* *			Heel bone mineral density	30598549; 28869591	Bone measurement	Heel bone mineral density (bmd)	0.003		**1.10E-15**		206496
rs11218343	11	121564878	0.96	C/T	*SORL1*	*1*.*09E-11*	*Overall (phase 3) (Jansen et al*, *2019)*	Alzheimer's disease (late onset)	30617256; 24162737	Neurological	Alzheimer's disease/dementia | illnesses of mother	-0.109761	0.8960485	1.37E-06	28507	331041
rs12590654	14	92472511	0.657	A/G	*SLC24A4*	1.65E-10	Overall (phase 3) (Jansen et al, 2019)	Alzheimer's disease (late onset)	30617256; 24162737	Neurological	Alzheimer's disease/dementia | illnesses of mother	0.035	1.036	1.25E-04	28507	331041
					* *						College or university degree | qualifications	-0.022	0.979	4.13E-05	115981	357549
rs442495	15	58730416	0.68	C/T	*ADAM10*	*1*.*31E-09*	*Overall (phase 3) (Jansen et al*, *2019)*	Family history of Alzheimer's disease	30617256; 29777097	Neurological						
					* *	* *	* *			Hematological measurement	Eosinophill count	-0.008636		4.71E-06		349856
					* *	* *	* *				Age started wearing glasses or contact lenses	0.202922		7.44E-06		310992
rs117618017	15	63277703	0.124	C/T	*APH1B*	3.35E-08	Overall (phase 3) (Jansen et al, 2019)	Family history of Alzheimer's disease	30617256; 29777097	Neurological						
					* *			Mean platelet volume	27863252	Autoimmune disease biomarker	Mean platelet (thrombocyte) volume	-0.040		**1.32E-27**		350470
					* *			Platelet count	27863252	Hematological measurement	Platelet count	1.305		**7.01E-11**		350474
					* *			Platelet distribution width	27863252		Platelet distribution width	-0.008		7.46E-06		350470
rs59735493	16	31121779	0.70	A/G	*KAT8*	3.98E-08	Overall (phase 3) (Jansen et al, 2019)	Family history of Alzheimer's disease	30617256; 29777097	Neurological						
					* *	* *	* *			Anthropometric measurement	Waist circumference	-0.218462		**1.91E-12**		360564
					* *	* *	* *			Anthropometric measurement	Hip circumference	-0.167485		**2.19E-12**		360521
					* *	* *	* *			Anthropometric measurement	Trunk fat mass	-0.090443		**2.15E-11**		354597
					* *	* *	* *			Anthropometric measurement	Whole body fat mass	-0.161863		**2.38E-11**		354244
rs113260531	17	5235685	0.881	A/G	*SCIMP*	9.16E-10	Overall (phase 3) (Jansen et al, 2019)	Family history of Alzheimer's disease	30617256; 29777097; 28092683	Neurological	Alzheimer's disease/dementia | illnesses of father	-0.078	0.925	1.20E-05	15022	312666
					* *			White blood cell count	27863252	Hematological measurement	White blood cell (leukocyte) count	-0.041		**5.71E-08**		350470
					* *					Hematological measurement	Neutrophill count	-0.028		**7.12E-08**		349856
rs28394864	17	49373413	0.53	A/G	*ABI3*	1.87E-08	Overall (phase 3) (Jansen et al, 2019)	Alzheimer's disease (late onset)	30617256; 24162737; 30326945; 30705288	Neurological						
					* *					Cardiovascular	Hypertension^b^	-0.0476	0.9535151	**1.60E-13**	77977	408343
					* *					Cardiovascular	Ischemic heart disease^b^	-0.0529	0.9484749	**4.58E-09**	31355	408458
					* *			Coronary artery disease	29212778	Cardiovascular	Coronary atherosclerosis^b^	-0.0682	0.9340736	**6.47E-10**	20023	397126
										Cardiovascular	Angina | vascular/heart problems diagnosed by doctor	-0.077758	0.9251879	**6.51E-09**	11372	360420
					* *					Respiratory system	Asthma | blood clot, dvt, bronchitis, emphysema, asthma, rhinitis, eczema, allergy diagnosed by doctor	-0.054635	0.9468312	**1.77E-13**	41633	360527
					* *						Impedance of whole body	1.79524		**1.16E-29**		354795
					* *			Eosinophil counts	27863252	Hematological measurement	Eosinophill count	-0.015258		**6.74E-18**		349856
					* *					Anthropometric measurement	Standing height	0.147437		**7.32E-23**		360388
rs2632516	17	58331728	0.548	C/G	*BZRAP1-AS1*	1.42E-09	Case-control status (phase 1) (Jansen et al, 2019)	Alzheimer's disease (late onset)	30617256; 24162737	Neurological						
					* *			Cognitive performance	30038396	Neurological						
					* *			Monocyte count	27863252	Hematological measurement	Monocyte count	-0.006		**1.84E-31**		349856
					* *			Plateletcrit	27863252	Hematological measurement	Plateletcrit	-0.001		**7.32E-16**		350471
					* *			Platelet count	27863252	Hematological measurement	Platelet count	-0.952		**1.02E-11**		350474
rs8093731	18	31508995	0.01	C/T	*SUZ12P1*	4.63E-08	Case-control status (phase 1) (Jansen et al, 2019)	Alzheimer's disease (late onset)	30617256; 24162737	Neurological						
rs76726049	18	58522227	0.9863	C/T	*ALPK2*	3.30E-08	Overall (phase 3) (Jansen et al, 2019)	Family history of Alzheimer's disease	30617256; 29777097	Neurological	Alzheimer's disease/dementia | illnesses of mother	-0.136	0.873	2.17E-04	28507	331041
rs76320948	19	45738583	0.96	C/T	*AC074212*.*3*	4.64E-08	Overall (phase 3) (Jansen et al, 2019)	Alzheimer's disease (late onset)	30617256; 24162737	Neurological	Alzheimer's disease^b^	0.64	1.8964809	5.96E-04	404	402787
rs6014724	20	56423488	0.0905	A/G	*CASS4*	6.56E-10	Overall (phase 3) (Jansen et al, 2019)	Alzheimer's disease (late onset)	30617256; 24162737	Neurological	Alzheimer's disease/dementia | illnesses of mother	-0.054	0.947	3.82E-04	28507	331041
rs7185636	16	19796841	0.84	C/T	*IQCK*	5.30E-08	Overall (Kunkle et al, 2019)	Alzheimer's disease (late onset) and AD-by-proxy	30820047	Neurological						
					* *						Impedance of whole body	-1.84821		**5.69E-19**		354795
					* *						Comparative body size at age 10	0.0179134		**2.67E-17**		354996
					* *					Anthropometric measurement	Body mass index (bmi)	0.0766973		**1.77E-07**		359983
rs2830500	21	26784537	0.686	A/C	*ADAMTS1*	2.60E-08	Overall (Kunkle et al, 2019)	Alzheimer's disease (late onset) and AD-by-proxy	30820047	Neurological						
rs114812713	6	41066261	0.98	C/G	*OARD1*	2.10E-13	Overall (Kunkle et al, 2019)	Alzheimer's disease (late onset) and AD-by-proxy	30820047	Neurological						
rs62039712	16	79321960	0.889	A/G	*WWOX*	3.70E-08	Overall (Kunkle et al, 2019)	Alzheimer's disease (late onset) and AD-by-proxy	30820047	Neurological						
rs138190086	17	63460787	0.98	A/G	*ACE*	7.50E-09	Overall stage 1 + stage 2 (Kunkle et al, 2019)	Alzheimer's disease (late onset) and AD-by-proxy	30820047	Neurological						
rs17125924	14	52924962	0.0954	A/G	*FERMT2*	1.40E-09	Overall stage 1 + stage 2 (Kunkle et al, 2019)	Alzheimer's disease (late onset) and AD-by-proxy	30820047	Neurological						
rs59685680	15	50709337	0.78	G/T	*SPPL2A*	7.32E-09	Combined (Liu et al, 2017)	AD-by-proxy	28092683	Neurological						
					* *			Neutrophil count	27863252	Hematological measurement	Neutrophill count	0.0270774		**1.43E-10**		349856
					* *			White blood cell count	27863252	Hematological measurement	White blood cell (leukocyte) count	0.0362551		**2.78E-09**		350470
rs2074612	5	140335105	0.455	C/T	*HBEGF*	8.00E-09	Combined (Liu et al, 2017)	AD-by-proxy	28092683	Neurological						
					* *			Cognitive performance	30038396	Cognitive function measurement						
					* *			Intelligence	29942086	Biological process						
					* *						Impedance of whole body	0.988		**4.97E-10**		354795
rs7384878	7	100334426	0.69	C/T	*PILRA*	1.30E-10	UK Biobank paternal and maternal meta-analysis	AD-by-proxy	29777097	Neurological	Alzheimer's disease/dementia | illnesses of mother	0.0440285	1.0450121	1.96E-06	28507	331041
					* *					Neurological	Alzheimer's disease/dementia | illnesses of father	0.05056	1.05186	4.98E-05	15022	312666
rs3845261	17	5104941	0.347	C/T	*ZNF232*	4.00E-08	UK Biobank paternal and maternal meta-analysis	AD-by-proxy	29777097	Neurological	Alzheimer's disease/dementia | illnesses of mother	0.029	1.030	1.30E-03	28507	331041
					* *			White blood cell count	27863252	Hematological measurement	White blood cell (leukocyte) count	0.021		4.24E-05		350470
rs72824905	16	81908423	0.01	C/G	*PLCG2*	5.38E-10	Sims et al., 2017	Alzheimer's disease (late onset)	28714976	Neurological						

P-values are in bold font if passing the PheWAS multiple testing correction threshold of 2.32x10^-7^ in UKB cohort.

*chromosomal position based on genome build GRCh38 coordinate.

**The Alleles column describes the two possible alleles at the variant location, listed in alphabetical order. In this study, the first allele will be called "A allele" and the second allele will be called the "B allele"; effect (β): The effect size, ln(Odds Ratio [OR]) for binary traits, defined per copy of the B allele.

*** Highlighted associations do not necessarily pass genome wide significance threshold in all PubMed ID citations.

Freq_B_ is frequency of B allele in Non-Finnish European from gnomAD.

^a^Analysis result was based on UKB Neale analysis accessed November 2019

^b^UKB SAIGE, while the rest of UKB results reported are based on UKB Neale v2 analysis accessed July 3, 2020.

*HLA-DRB1* variant rs6931277 associated with AD was also associated with diseases with an immune-related etiology such as ulcerative colitis (UC, *p* = 2.23 x10^-10^), self-reported rheumatoid arthritis (RA, *p* = 2.30 x 10^−130^), celiac disease (CeD, *p* = 5.99 x10^-54^), self-reported asthma (*p* = 8.57 x 10^−68^), self-reported multiple sclerosis (*p* = 1.22 x 10^−13^), Type 1 diabetes (*p* = 1.55 x10^-60^) in the UKB cohort. In addition, *SPPL2A*, *CD33*, *SCIMP*, *ADAMTS4*, *APH1B*, *BZRAP1-AS1*, *ZNF232*, *GRN*, and *CD2AP* variants were associated with mean platelet (thrombocyte) volume (an autoimmune disease biomarker), neutrophill count, monocyte count/percentage, and/or white blood cell (leukocyte) count. The significance of these associations is unknown given the large study sample size and the small effect size, but the directionality in most cases (if not all) is consistent with reported in the literature in a study with a large sample size (>160,000 subjects) [53]. *ABI3* variant was also associated with asthma in the UKB cohort (*p* = 1.77 x 10^−13^). The association results from the 23andMe cohort for selected immune-related conditions are listed in S5 Table. In the UKB cohort, *CD33* variant rs3865444 had nominal association with asthma (*p* = 2.84 x 10^−4^), while *CD2AP* variant rs9349407 had nominal association with UC (*p* = 0.0004) and *PICALM* variant rs3851179 had nominal association with hay fever or allergic rhinitis (*p* = 4.42 x 10^−4^).

FTD variant rs646776 is known to be associated with LDL-cholesterol levels, cardiovascular diseases, and plasma progranulin levels. Our PheWAS analysis (Fig 2) replicated the associations of rs646776 with metabolic traits (high cholesterol or taking drugs to lower cholesterol *p* = 2.74 x 10^−207^; high cholesterol, *p* = 1.35 x 10^−185^), low high-density lipoproteins (HDL, *p* = 3.37 x 10^−47^), cardiovascular traits (heart attack (*p* = 1.37 x 10^−7^), CAD (*p* = 1.79 x 10^−14^), angina (*p* = 2.34 x 10^−6^). Rs646776 was also significantly associated with longevity traits (heart metabolic disease in old people, healthy old) and height (*p* = 1.80 x 10^−9^) and suggestively associated with coronary bypass surgery (*p* = 5.24 x 10^−5^, FDR = 0.004), aortic stenosis (*p* = 0.0001, FDR = 0.01) and angioplasty (*p* = 0.0007, FDR = 0.04). rs5848 had a suggestive association with Parkinson’s disease in both the 23andMe cohort (*p* = 1.38 x 10^−5^) and the UKB cohort (*p* = 3.89 x 10^−3^).

In addition to *APOE* variants and rs646776, *ABI3* variant rs28394864 was also associated with cardiovascular traits, such as hypertension (*p* = 1.60 x 10^−13^), ischemic heart disease (*p* = 4.58 x 10^−9^), coronary atherosclerosis (*p* = 6.47 x 10^−10^), and angina (*p* = 6.51 x 10^−9^). Despite *CLU* and *ABCA7* are both implicated in cholesterol metabolism [22], neither the *CLU* variant nor the *ABCA7* variant was strongly associated with metabolic/cardiovascular traits in the PheWAS analyses in either the 23andMe cohort or the UKB cohort despite the fairly substantial sample size for those traits in both cohorts.

The PheWAS results for the UKB cohort are available in S6 Table (Neale v1) and S7 Table (Neale v2 and UKB SAIGE).

### No significant genetic correlation between PheWAS traits and AD

Despite that multiple traits were associated with the same individual variants in PheWAS analysis, there was no significant genetic correlation among these traits (e.g. LDL/HDL cholesterol, Type 2 Diabetes, CAD, CeD, RA, UC, multiple sclerosis, and BMI) and AD at the genome level (S8 Table). As positive controls, IGAP AD [21] and UKB trait (Neale v1) Illnesses of mother: AD/dementia showed significant genetic correlation with Jansen et al AD results [30] (r_g_ = 0.901, *p* = 3.10 x 10^−13^; r_g_ = 0.63, *p* = 1.09 x 10^−6^, respectively).

### A causal role of cholesterol on AD revealed by MR analysis

Among the significant PheWAS traits associated with the AD/FTD disease variants, a set of genetic variant instruments from MR Base for BMI [54, 55], T2DM [56–58], lipid traits including HDL cholesterol, LDL cholesterol, and total cholesterol [59], CAD [60, 61], extreme height [55], parental attained age [62], traits defined from UK Biobank such as “reason for glasses/contact lenses: For short-sightedness i.e. only or mainly for distance viewing such as driving, cinema, etc. (called 'myopia')”, and “non-cancer illness code self-reported: angina” analyses by the Neale Lab v1), RA [63], Inflammatory bowel disease (UC and Crohn's disease (CD)) [64, 65], CeD [66], multiple sclerosis (MS) [67, 68] were obtained. When treating AD as outcome and using *p* < 5x10^-8^ to select variants as instrument variables, the MR Egger intercept test suggested a directional horizontal pleiotropy for extreme height (Egger intercept *p* = 0.009), total cholesterol (*p* = 0.02), RA (*p* = 0.02) and parents’ age at death (Egger intercept *p* = 0.02). MR Egger analysis suggested that metabolic traits (e.g. LDL cholesterol (*p* = 4.7 x 10^−4^) and total cholesterol (*p* = 9.8 x 10^−5^)) and RA had protective effect on AD with higher level of LDL or total cholesterol increasing the risk of AD and having RA reduce the risk of AD (S9 Table).

Conversely, genetic variant instrument for AD [30] suggested that AD possibly had causal effect on MS (*p* = 0.0001 using inverse variance weighted method) and coronary heart disease (*p* = 0.003 using MR Egger method, S9 Table). For FTD, the results may be inconclusive due to few SNPs were used in the instrument variable and the SNPs chosen were suggestively significant from the GWAS with smaller sample size. These MR tests would be still significant after correcting for the number of traits tested (n = 15, p < 0.05/15 ~ 0.003). A full list of MR results is listed in S9 Table.

## Discussion

The PheWAS study showed that both *APOE* variants defining **ε**2/**ε**3/**ε**4 alleles, *ABI3* variant rs28394864, and rs646776 had significant associations with metabolic/cardiovascular and/or longevity traits. *APOE* variants were additionally significantly associated with neurological traits. *HLA- DRB1* variant was associated with immune-related traits. Both *APOE* variants and *CLU* variant were significantly associated with eye phenotypes. The associations of *ABI3* variant rs28394864 with cardiovascular traits (hypertension, ischemic heart disease, coronary atherosclerosis, angina), and asthma are novel findings from this study.

The novel finding of PheWAS associations of *ABI3* variant is of most interest. Rare variant (rs616338, p.Ser209Phe, *p* = 4.56 × 10^−10^, OR = 1.43, MAF_cases_ = 0.011, MAF_controls_ = 0.008) in *ABI3* was previously reported, and *ABI3* is specifically expressed in microglia (S2 Fig, similar expression pattern in human compared to other AD genes implicated by human genetics including *TREM2*, *HLA-DRB1*, *PLCG2*, *SORL1*, *SCIMP*, and *MS4A6A*) and thought to play a role in microglia-mediated innate immunity in AD [69]. Given its role in immune response, the PheWAS association with asthma is not completely unexpected, and its association with cardiovascular traits might reflect the role of immune dysregulation on those disease processes.

The observed PheWAS associations of *APOE* variants with metabolic/cardiovascular traits are not surprising. While vascular and metabolic risk factors such as hypertension, hyperlipidemia /hypercholesterolemia, hyperinsulinemia, and obesity at midlife, diabetes mellitus (DM), and cardiovascular and cerebrovascular diseases (including stroke, clinically silent brain infarcts and cerebral microvascular lesions) are generally thought to increase the risk of dementia and AD [70–73], the directional impact of a factor could be age-dependent, for example, hypertension, obesity and hypercholesterolemia are risk factors at middle age (<65 years) for late-life dementia and AD, but protective late in life (age >75 years) [74]. It seems to be odd that AD patient had a lower risk of developing CAD [73], but it is consistent with a meta-analysis [72] and this meta-analysis also reported that metabolic syndrome decreases the risk of AD. In the MR analysis from this study, AD increased the risk of CAD (S9 Table), but this result was supported by MR Egger method only. Taking age into consideration may help better delineate the relationship. Furthermore, several cardiovascular risk factors demonstrated associations with more rapid cognitive decline as expected, however it was also reported that recent or active hypertension and hypercholesterolemia were associated with slower cognitive decline for AD patients [75]. These epidemiology studies suggested that it appears to be a complex interplay between AD and metabolic/cardiovascular risk factors and conditions, and the occasionally contradictory findings may be due to age of the population, sampling biases and/or other confounding factors. Nevertheless, the Finnish Geriatric Intervention Study to Prevent Cognitive Impairment and Disability (FINGER) study demonstrated that multidomain intervention (diet, exercise, cognitive training, vascular risk monitoring) had beneficiary effect on the primary outcome, i.e. change in cognition as measured through comprehensive neuropsychological test battery (NTB) in an at-risk elderly population (aged 60–77) with CAIDE (Cardiovascular Risk Factors, Aging and Dementia) Dementia Risk Score of at least 6 points and cognition at mean level or slightly lower than expected for his/her age group, suggesting targeting modifiable vascular and lifestyle-related risk factors could improve or maintain cognitive functioning [76]. The PheWAS analysis suggested the minor allele of rs7412 (defining ε2 allele), a known protective allele for AD (OR = 0.74), was also a protective allele for having high cholesterol, low HDL, having heart metabolic disease or CAD. Similarly, the minor allele of rs429358 (defining ε4 allele), a known risk allele for AD (OR = 2.17), was also a risk allele for having high cholesterol, low HDL, having heart metabolic disease or CAD. Our MR analysis demonstrated that LDL and total cholesterol had a causal relationship to the development of AD using MR Egger. This MR result is however sensitive to the MR methods used as other methods such as weighted mode, weighted median, or simple mode (not pre-specified analyses) did not provide evidence or only provide suggestive evidence for the causal effect of LDL on AD. A recent MR analysis on 24 potentially modifiable risk factors [77] concluded that genetically predicted cardiometabolic factors were not associated with AD as there was no evidence of causal relationship after excluding one pleiotropic genetic variant (not disclosed in the publication) near the *APOE* gene (also near *APOC1* and *TOMM40* genes). The evidence we obtained was far weaker than that reported by Larsson et al., for all variants [77]. Despite there were few SNPs driving the causal evidence in single variant analysis, leave-one-analysis did not differ substantially from the analysis including all variants for LDL trait except rs7412 (S1 Fig). This study opted to report the findings using the inverse variance weighted method (when Egger intercept is not significant) as also adopted by Howard et al. [78], where a minimal of 30 SNPs used in instrument variable was also imposed, or MR Egger regression results (when the intercept is significant). We did not filter out analysis with less than 30 variants. Both compromises are limitations in this study thus those results shall be interpreted with caution. In addition, both IVW and MR Egger methods do not protect against the violation of the MR assumption when the pleiotropic effects act via a confounder of the exposure-outcome association [49].

The observed PheWAS associations of rs646776 variant with metabolic/cardiovascular traits are also not surprising. SNP rs646776 was reported to be robustly associated with low-density lipoprotein cholesterol (LDL-C, *p* = 3 × 10^−29^) with each copy of the minor allele decreasing LDL cholesterol concentrations by ~5–8 mg/dl [79]. The association was strengthened in a meta-analysis of ~100,000 individuals of European descent for LDL-C (*p* = 5x10^-169^) and was also detected for total cholesterol (*p* = 7x10^-130^) [80]. However, which gene is the causative gene for rs646776 effect is less clear despite it was selected to be included in the PheWAS analysis based on the association with plasma progranulin levels. Rs646776 at the 1p13 locus was also strongly associated with transcript levels of three neighboring genes: sortilin (*SORT1*) (*p* = 3 × 10^−26^), cadherin EGF LAG seven-pass G-type receptor 2 (*CELSR2*) (*p* = 2 × 10^−12^) and proline and serine rich coiled-coil 1 (*PSRC1*) (*p* = 3 × 10^−12^) [79]. The conditional analysis suggested that *SORT1* eQTL effect might be the dominant effect [79]. Rs599839, a SNP in LD with rs646776, was also reported to be associated with CAD [81]. The minor allele conferring lower level of LDL cholesterol also conferred lower risk of CAD. Rs646776 was also identified in a bivariate analysis to be associated with circulating IGF-I and IGF-binding protein-3 (IGFBP-3) (*p* = 6.87 x10^-9^) in a meta-analysis of 21 studies including 30,884 adults of European ancestry [82]. The growth hormone/insulin-like growth factor (IGF) axis can be manipulated in animal models to promote longevity. IGF related proteins including IGF-I and IGFBP-3 have also been implicated in risk of human diseases including cardiovascular diseases and diabetes. This is particularly interesting given the observation that rs646776 is associated with longevity in the PheWAS analysis.

It is surprising and puzzling to see the effect of AD variants on multiple eye phenotypes especially myopia that have onset in early childhood or teens. The association with age related macular degeneration (AMD) was reported previously [83]. The AMD association is interesting because the histopathological hallmark of AMD is amyloid-β (Aβ) in optic never drusen [84]. Drusen of the macula are very small yellow and white spots that appear in one of the layers of the retina named Bruch’s membrane and are remnant nondegradable proteins and lipids (lipofuscin), which is the earliest visible sign of dry macular degeneration. In addition to the amyloid phenotype, AMD and AD also share other common histologic feature such as vitronectin accumulation and immunologic features such as increased oxidative stress, and apolipoprotein and complement activation pathways [85]. The common etiopathogenetic and morphological manifestations of AD and age-related eye diseases in amyloid genesis may have a broader implication in understanding the disease mechanism, identifying new biomarkers and treatment [86]. A recent study showed that the soft drusen area in amyloid-positive patients was significantly larger than that in amyloid-negative patients [87]. Ocular and visual information processing deficit were other possible biomarkers for AD [88]. Recently it was also reported that thinner retinal nerve fiber layer is associated with an increased risk of dementia including AD, suggesting that retinal neurodegeneration may serve as a preclinical biomarker for dementia [89]. Risk variant for AD rs429358 in our PheWAS results had a protective effect for AMD and blindness (*p* = 9.6 x 10^−5^, FDR = 0.004), perhaps reflecting the equilibrium of Aβ in brain vs. retina like the situation between brain vs. CSF. A variety of other visual problems reported in patients with AD have been reviewed in details [90] including loss of visual acuity (VA), color vision and visual fields; changes in pupillary response to mydriatics, defects in fixation and in smooth and saccadic eye movements; changes in contrast sensitivity and in visual evoked potentials (VEP); and disturbances of complex visual functions, though they have not been studied as a risk factor of AD or outcome of having AD. In the MR analysis, we cannot directly test the causal relationship between AMD despite the GWAS with large sample size is available due to standard error of odds ratio was not reported in the paper [83].

Subjects with AD risk variant rs1113600 in *CLU* gene had a higher chance of being nearsighted, while subjects with AD risk variant rs429358 in *APOE* gene had a lower chance of being nearsighted. It was reported that wearing reading glasses correlated significantly with high mini-mental state examination for the visually impaired (MMSE-blind) after adjustment for sex and age (OR  =  2.14, 95% CI  =  1.16–3.97, *p*  =  0.016), but reached borderline significance after adjustment for education [91]. There was a trend toward correlation between myopia and better MMSE-blind (r  =  -0.123, *p*  =  0.09, Pearson correlation) [91]. On the other hand, myopia may be a surrogate phenotype for intelligence (or education), as a genetic correlation between myopia and intelligence was shown in a small cohort of 1500 subject (*p* < 0.01) [92]. Larsson et al., suggested that genetically predicted educational attainment was significantly associated with AD per year of education completed (OR = 0.89, *p* = 2.4 × 10^−6^) and per unit increase in log odds of having completed college/university (OR = 0.74, *p* = 8.0 × 10^−5^), while intelligence had a suggestive association with AD (OR = 0.73, *p* = 0.01) [77]. Our MR analysis did not provide evidence on the causal relationship of myopia phenotype on AD. Furthermore, although genetic variants (rs429358 and rs1113600) are associated with multiple phenotypes, the associations are not necessarily independent of each other. In fact, MR Egger intercept test did not support the independent relationship except for height and a few other traits. Overall, the relationship and interpretation between traits seem to be complex and require further examination.

Other limitations of our study design also merit comment. The sample size for PheWAS varies from trait to trait depending on the prevalence rate of the trait and availability of data. For example, the cohort size for AD in the 23andMe database was not large in 2015 (~640 cases and ~158K controls) when the PheWAS analysis for the 23andMe cohort was performed, which is a limitation for this study especially for replicating the association with AD. This may explain why some of the known SNP associations with AD were not replicated or only had nominally significant association in the 23andMe cohort. Furthermore, FTD is not a self-reported question collected in the 23andMe database and therefore could not be tested in the PheWAS analysis. Even if this was included, the sample size would have been smaller than that for AD based on population prevalence rate. Similarly, the cohorts for CD (n~3,600), UC (n~6,200), bipolar (~9,700), and schizophrenia (~700) were limited in size. However, the cohort sizes for other psychiatric disorders (e.g. depression, anxiety and panic) were sufficiently large (>250K cases). The PheWAS 23andMe cohort size for AD used in this study was limited, and therefore only *APOE* variants, the loci with the largest effect size, were confirmed to be associated with neurological traits. The sample size for a specific trait shall be taken into consideration when interpreting the PheWAS results. PheWAS typically uses “light” phenotyping (based on self-reported as in the 23andMe and surveys deployed by UKB or based on diagnostic ICD codes or medication / procedure usage pattern), the stringency of phenotype is certainly not as good as clinical ascertained phenotype, but the tradeoff is the power to survey a large number of diverse phenotypes within a single study.

The nominal causal effect between immune-related traits (except multiple sclerosis and rheumatoid arthritis) and AD/FTD would have been insignificant if correcting for the expanded list of diseases and risk factors from MR Base tested. Some of the instrument variable used consisted of small number of SNPs and may have weaken the real causal effect if exist. The observation does not seem to be purely by chance especially in light of the report on immune related enrichment of FTD where they found up to 270-fold genetic enrichment between FTD and RA, up to 160-fold genetic enrichment between FTD and UC, and up to 175-fold genetic enrichment between FTD and CeD. Overall, the immune overlap seems to be common to both FTD and AD at the genome level (represented by genome wide significant SNPs used as an instrument variable for AD and other diseases/risk factors except FTD where a p-value threshold of 5 x 10^−6^ was used because of the smaller GWAS samples size), while there could still be specificity of neuroinflammation for risk variants in *CR1*, *CD33*, *CLU*, *ABCA7*, *TREM2*, *SORL1*, *MS4A6A*, *SPPL2A*, *SCIMP*, *PLCG2*, *ABI3*, and *HLA-DRB1*. Different MR analysis methods have different assumptions (which in reality do not always hold or even rarely hold) and power, the inference of the causal effect may be inconclusive or only suggestive unless the causal effect size is so huge that most of methods give unequivocal concordant results. Both IVW and MR Egger methods used in this study are vulnerable to false positives when the exposure and outcome traits are both affected by a heritable confounder [49]. Different exposure or outcome GWAS may also vary by study sample size, and number of variants with summary association statistics available (for outcome GWAS as this may limit the ability to leverage proxy SNPs in LD (default r^2^ > = 0.8) with the set of SNPs in the instrument variable) and impact the strength of instrument variable and the power of MR analysis. Future re-analysis when studies with larger sample size and more complete summary association statistics will be warranted to interrogate the causal relationship.

## Supporting information

S1 Table Genotype platform representation and imputed variant statistics in PheWAS. (XLSX)

S2 Table A list of 1234 phenotypes from the 23andMe cohort used for PheWAS analyses and sample size for each trait using the rs3865444 PheWAS results as an example. (XLSX)

S3 Table Detailed PheWAS results (FDR < 0.05) from the 23andMe cohort. (XLSX)

S4 Table A full list of detailed PheWAS results from the 23andMe cohort. (XLSX)

S5 Table Detailed results of PheWAS for immune-related traits. (XLSX)

S6 Table A full list of detailed results of PheWAS from the UKB cohort from Open Target Genetics (Neale v1) accessed in May 2019. (XLSX)

S7 Table A full list of detailed results of PheWAS from the UKB cohort from Open Target Genetics (Neale v2 and UKB SAIGE) accessed in July 2020. (XLSX)

S8 Table MR analysis for PheWAS traits (full results) and selected immune-related traits (*p* < 0.1). (XLSX)

S9 Table MR analysis for exposure = LDL and outcome = AD sensitivity analysis. (XLSX)

S1 Fig MR analysis on the LDL exposure and AD outcome. (DOCX)

S2 Fig Cell type specificity of *ABI3*, *SORL1*, *HLD-DRB1*, *MS4A6A*, *TREM2*, *PLCG2*, and *SCIMP*. (DOCX)
