# Supplementary material for: The association of clinical phenotypes to known AD/FTD genetic risk loci and their inter-relationship
Source: PLoS One. 2020 Nov 5;15(11):e0241552. doi: 10.1371/journal.pone.0241552 (PMC7644002; doi:10.1371/journal.pone.0241552)
Supplement: S1 Fig — (DOCX) [file pone.0241552.s010.docx]

**S1 Fig. MR analysis on the LDL exposure and AD outcome (a) single variant analysis; (b) leave one out analysis; (c) funnel plot. Genetic instrument variables are selected using p < 5x10^-8^.**

**(A)**

**
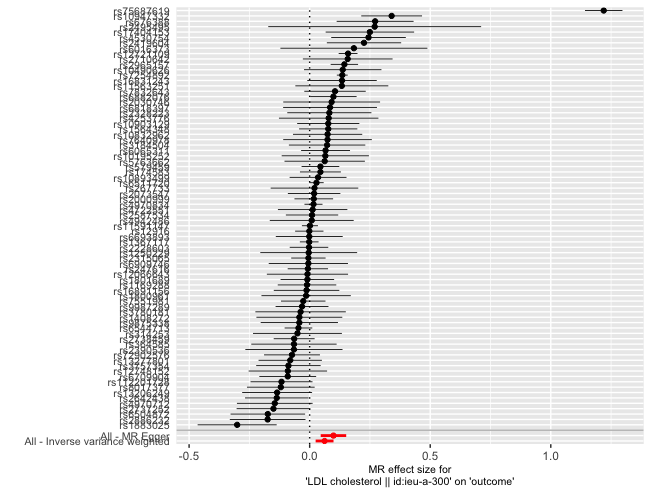
**

**(B)**

**
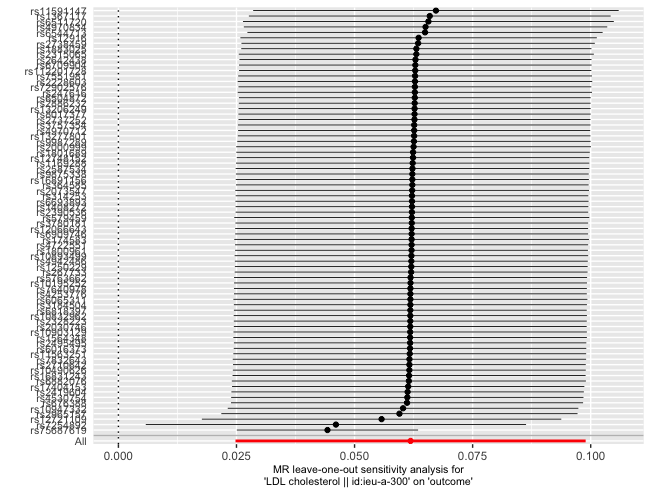
**

**(C) Funnel plot**

**
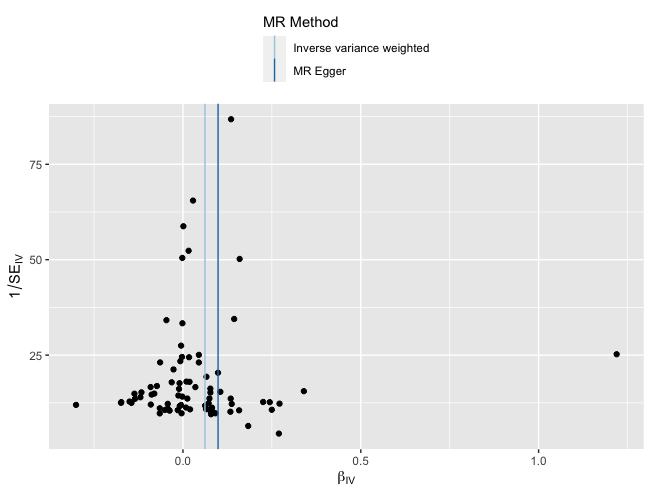
**
