# Supplementary material for: The association of clinical phenotypes to known AD/FTD genetic risk loci and their inter-relationship
Source: PLoS One. 2020 Nov 5;15(11):e0241552. doi: 10.1371/journal.pone.0241552 (PMC7644002; doi:10.1371/journal.pone.0241552)

**S2 Fig.** Cell type specificity of *ABI3 (A, B)**, SORL1 (C, D), HLD-DRB1* (E), *MS4A6A* (F, G), *TREM2* (H, I), *PLCG2* (J, K), *SCIMP* (L, M), *GRN* (N, O), *ADAM10* (P, Q), *ADAMTS4* (R, S), *CD2AP* (T, U), and *APH1B* (V, W) in mouse (A, C, F, H, J, L, N, P, R, T, V) and human (B, D, E, G, I, K, M, O, Q, S, U, W) (source: <https://www.brainrnaseq.org/>)

(A)


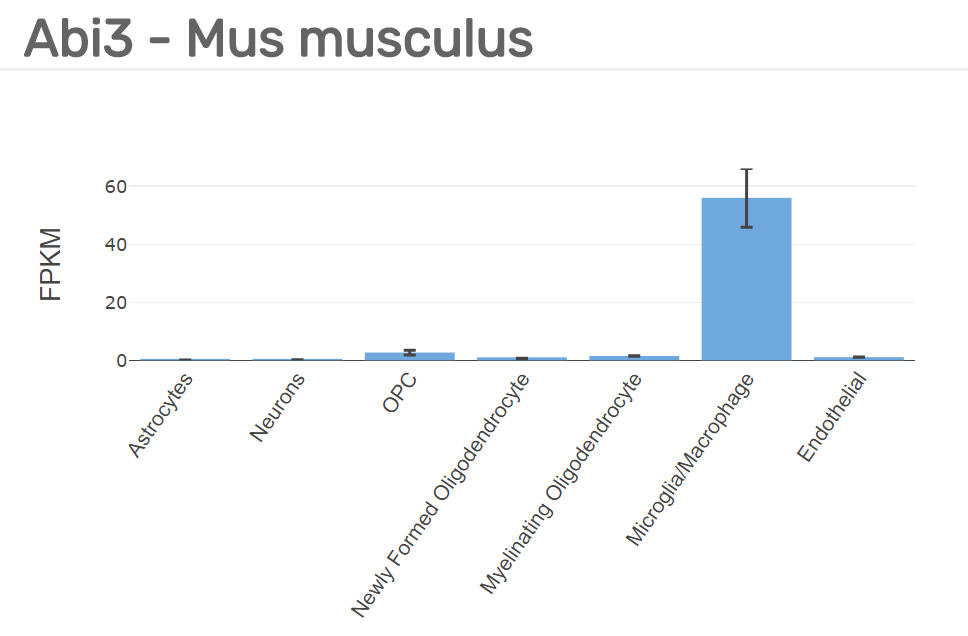


(B)


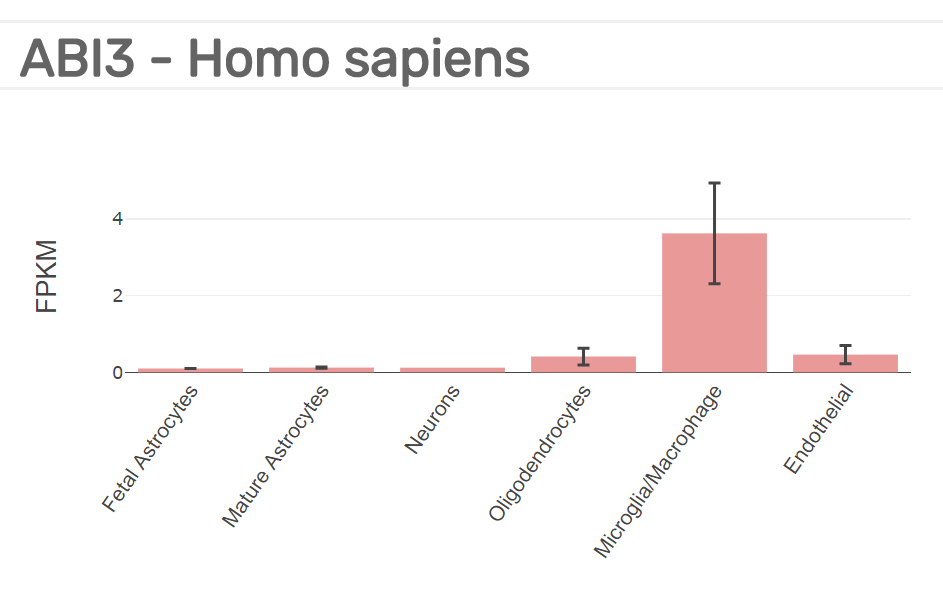


(C)


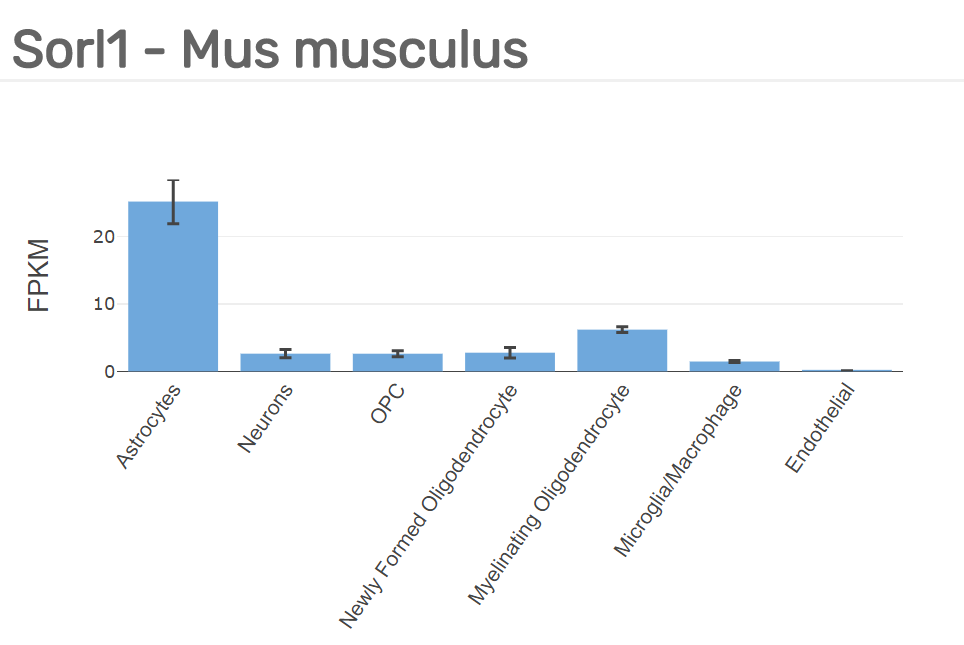


(D)


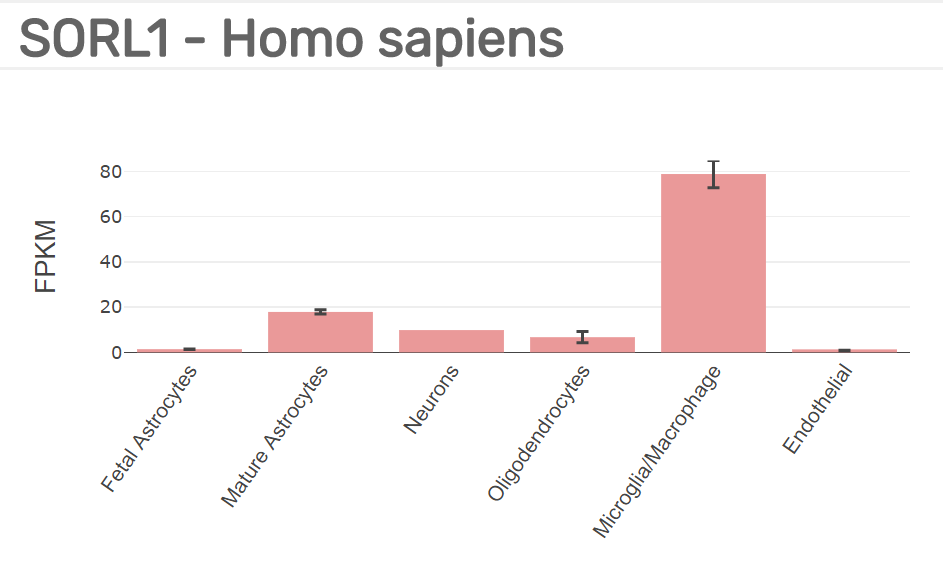


(E)


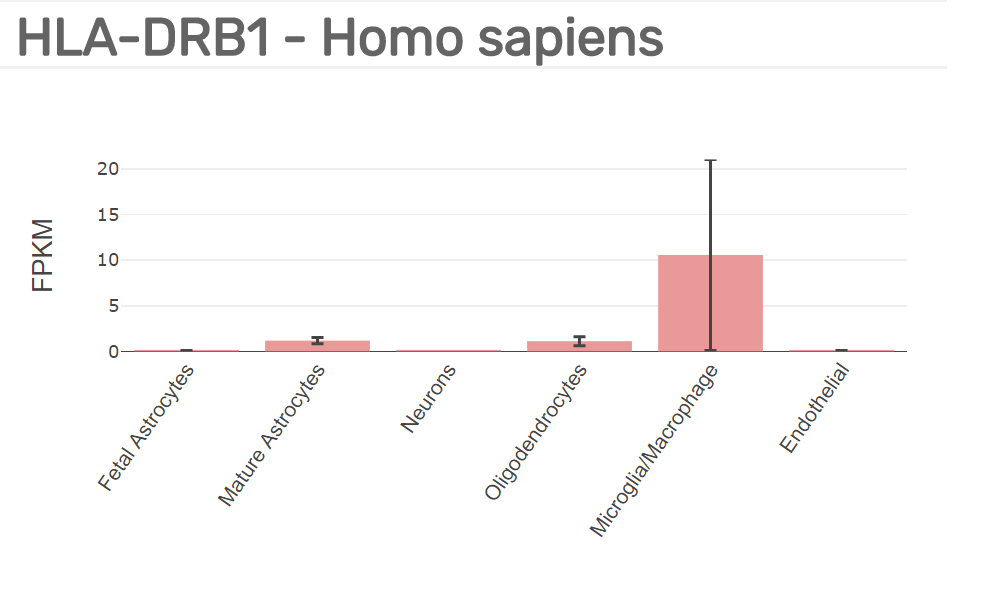


(F)


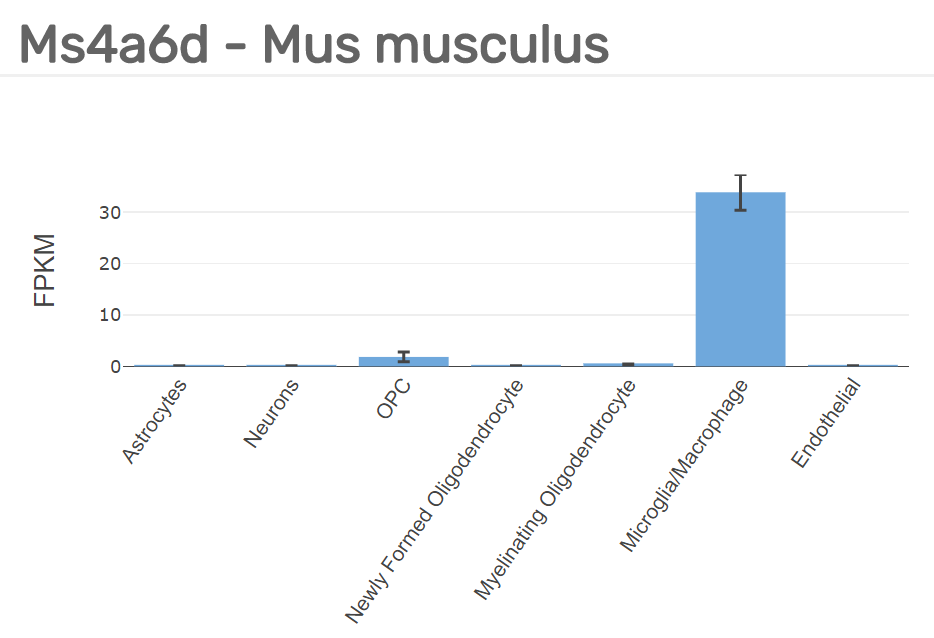


(G)


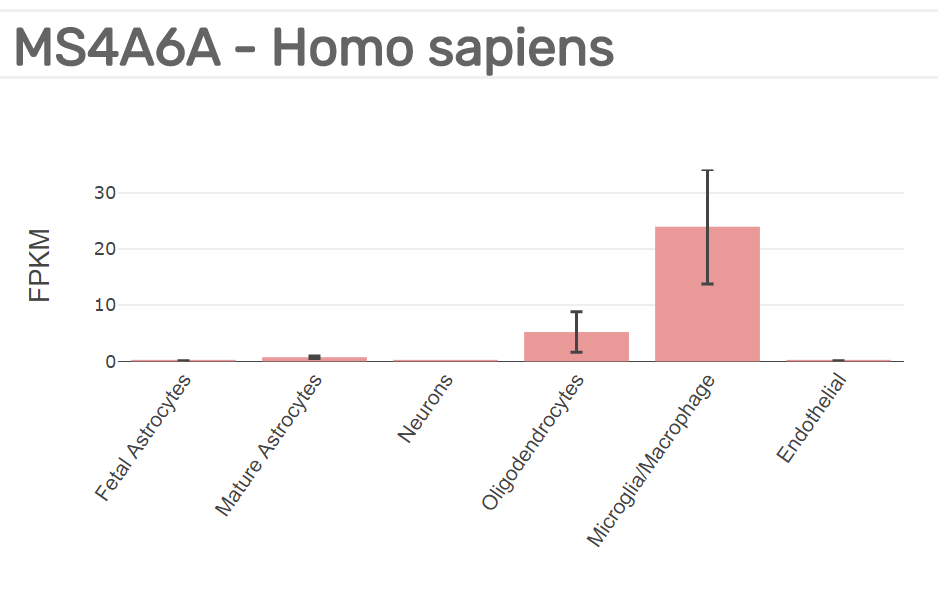


(H)


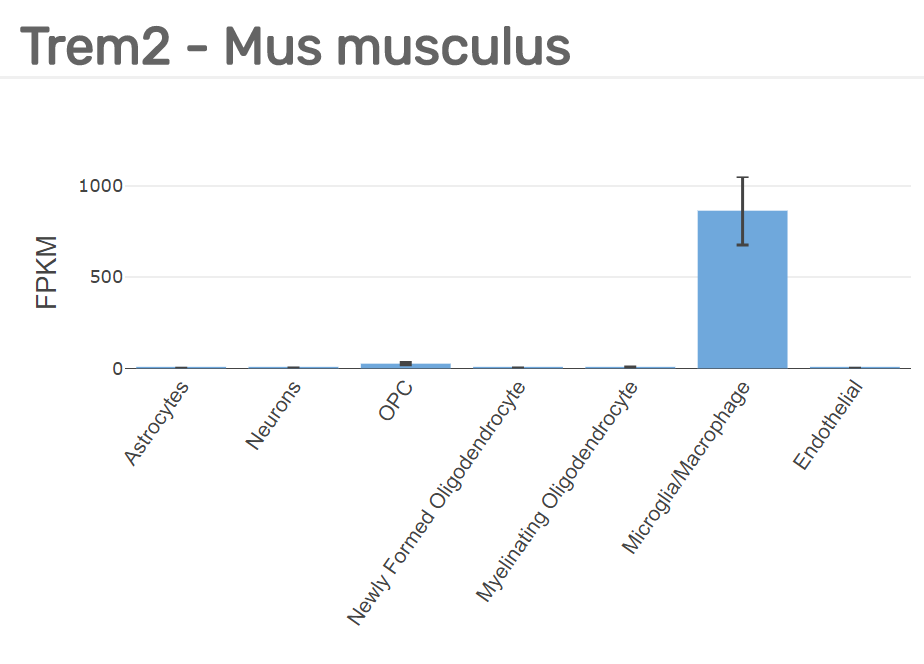


(I)


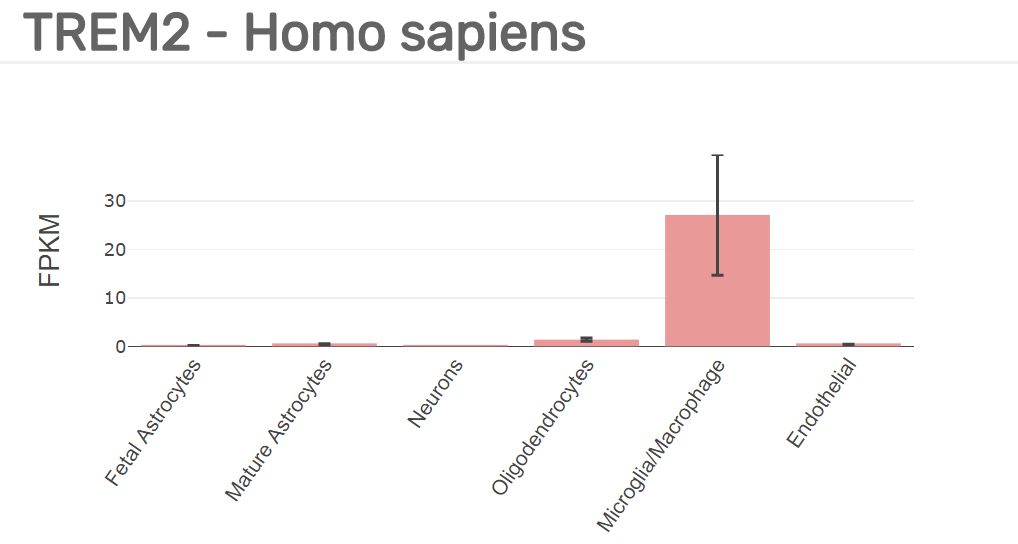


(J)


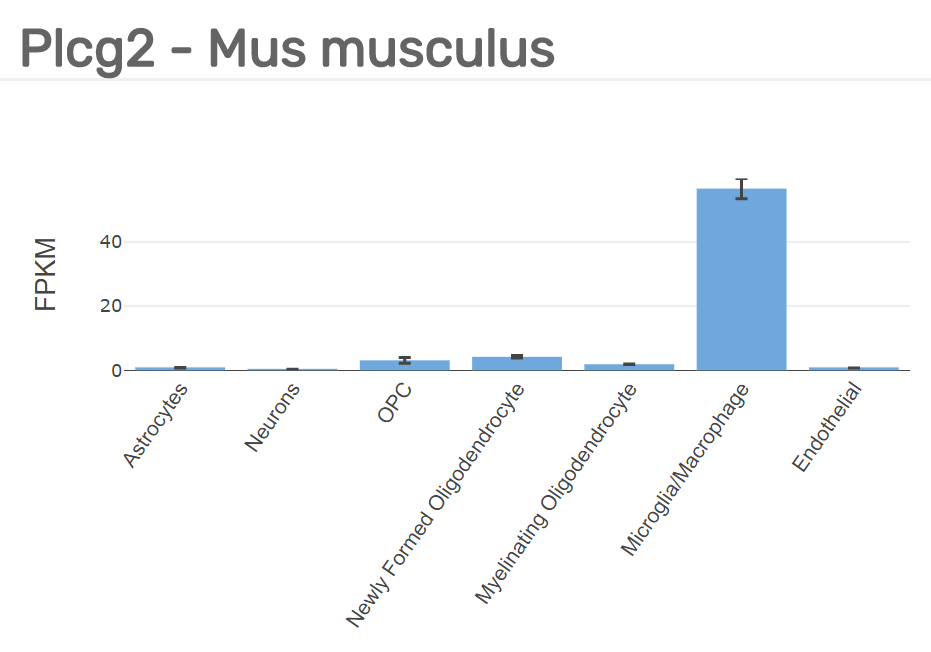


(K)


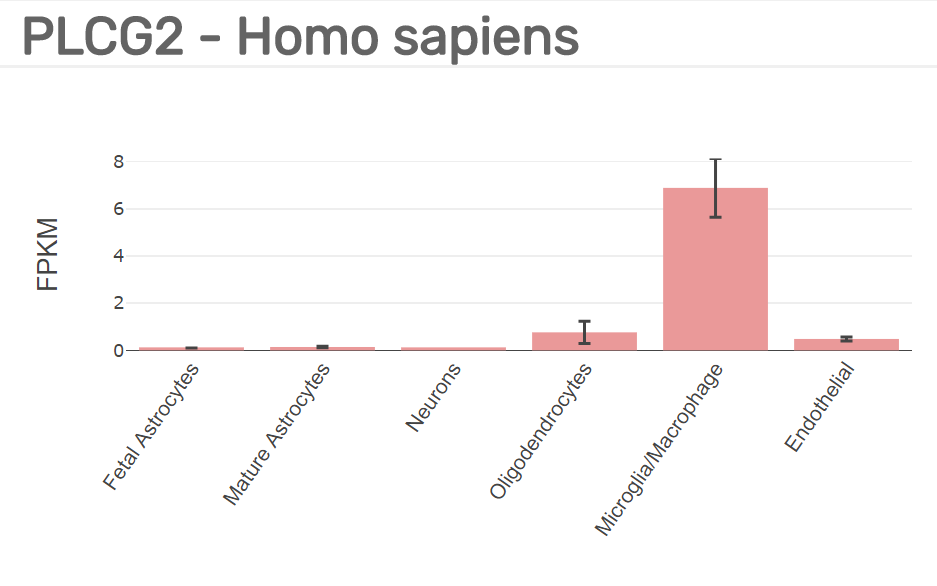


(L)


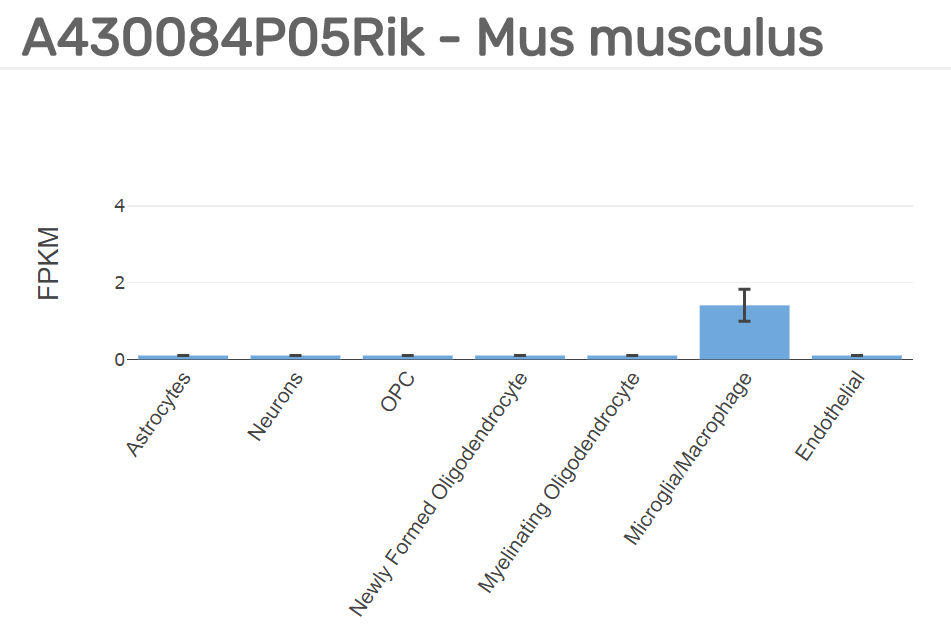


(M)


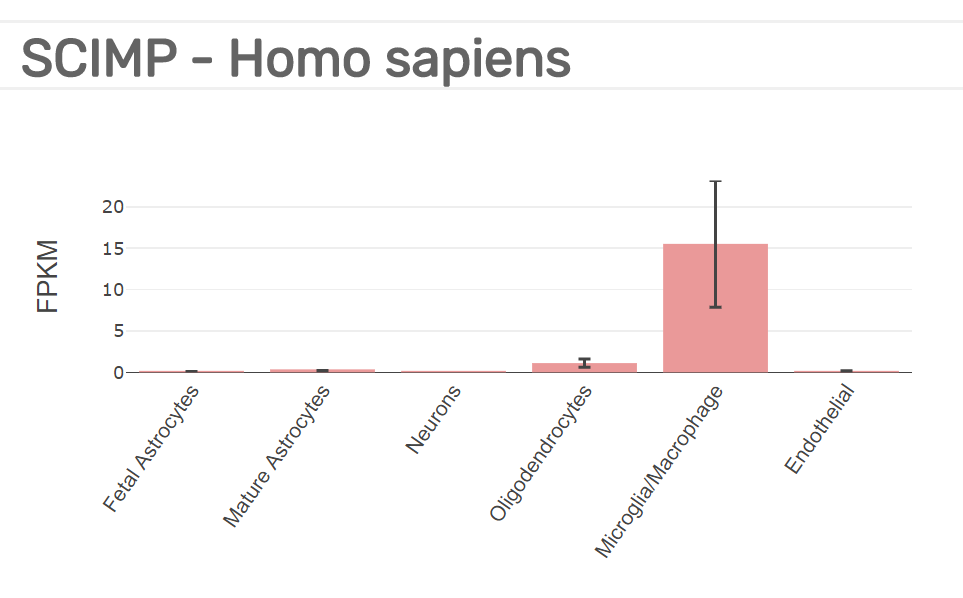


(N)


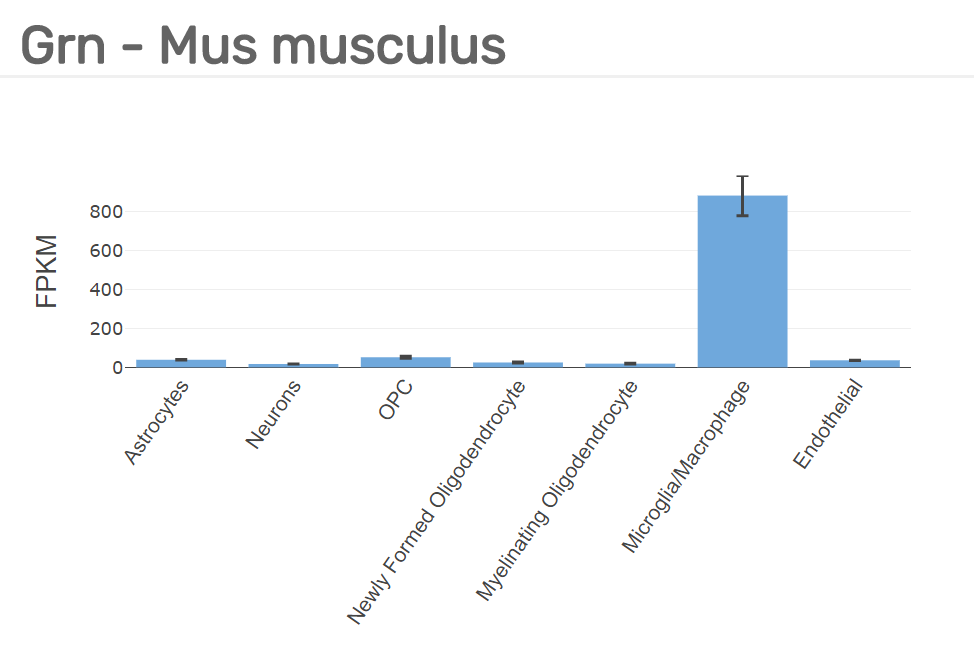


(O)


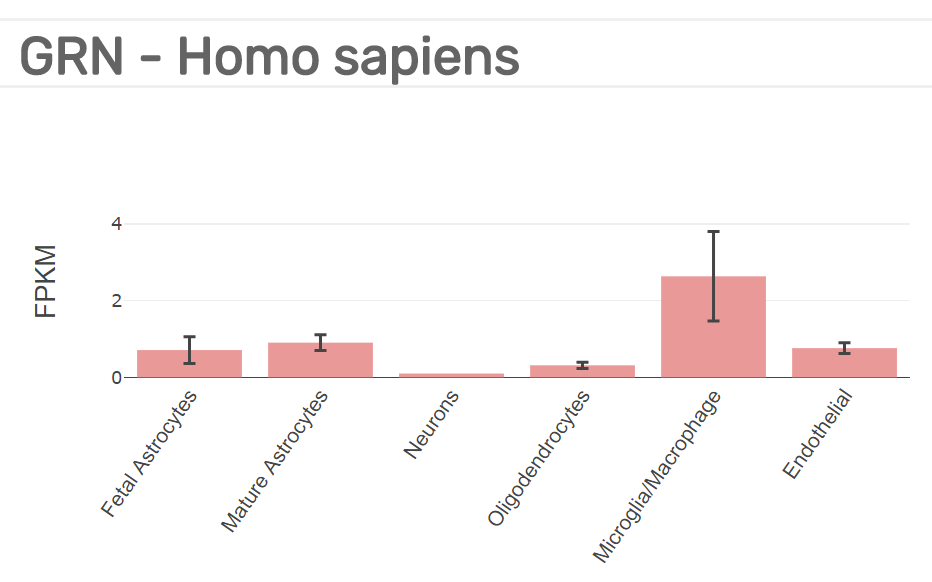


(P)


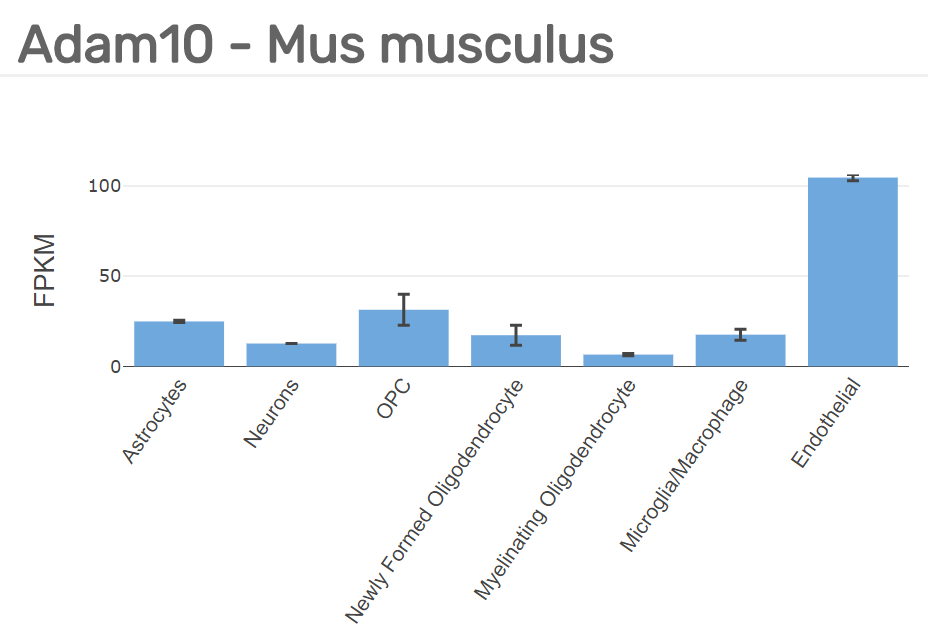


(Q)


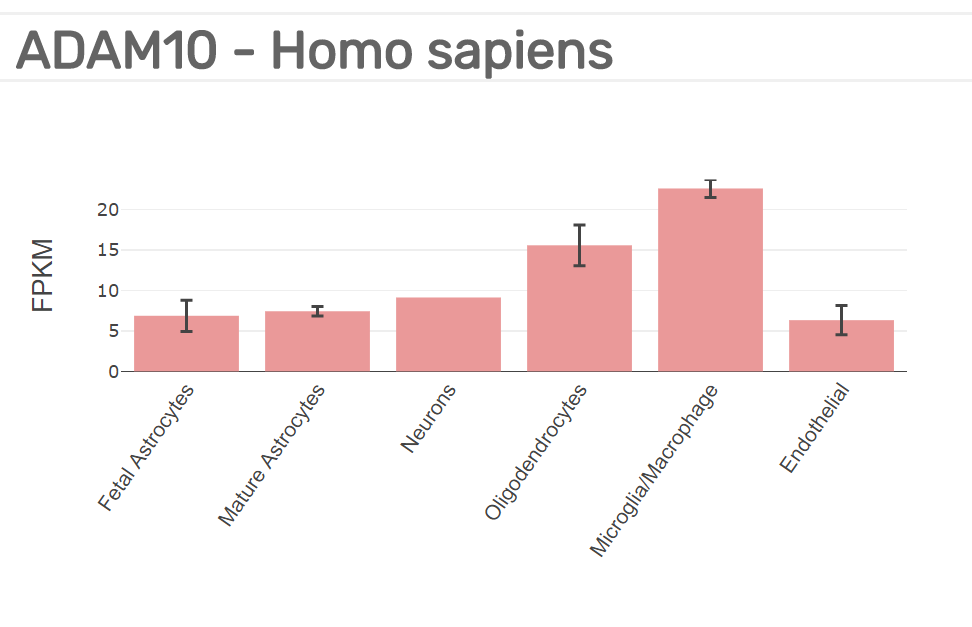


(Q)


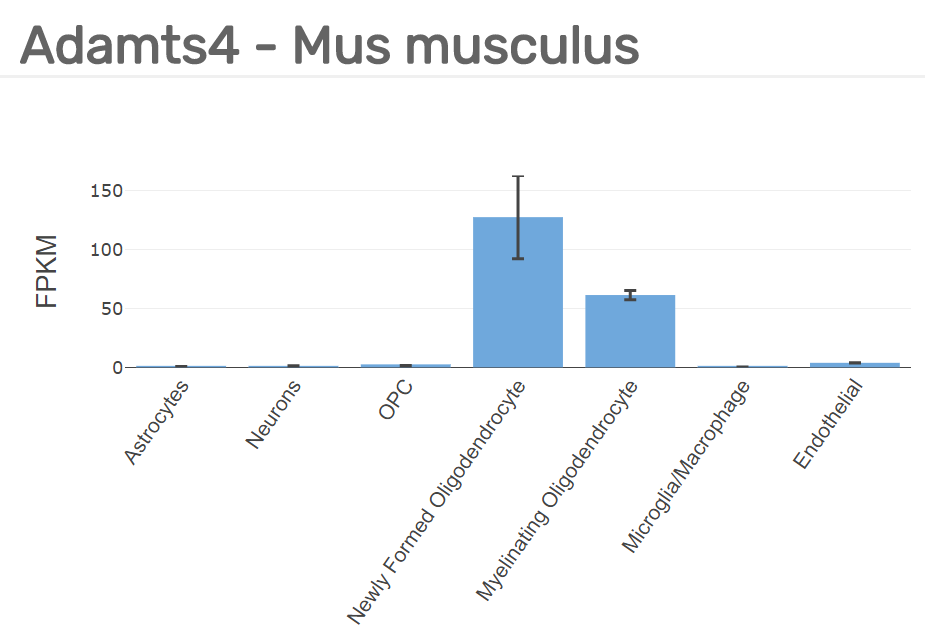


(S)


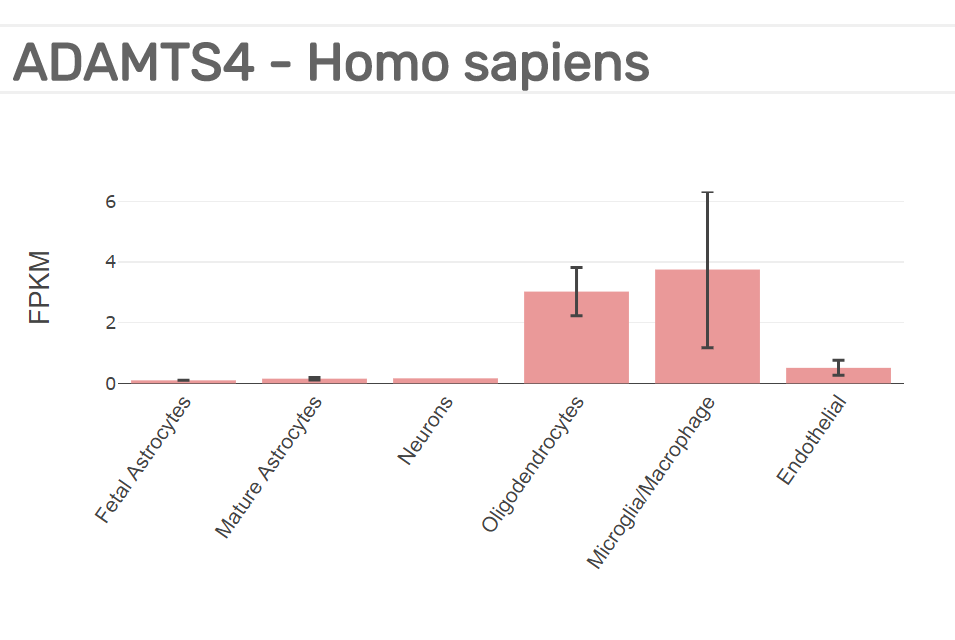


(T)


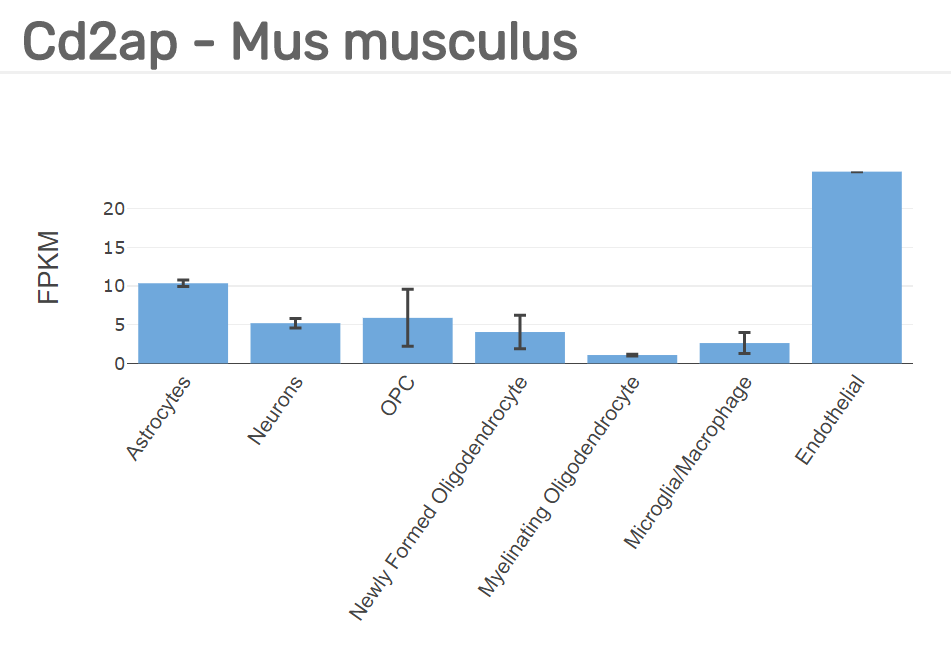


(U)


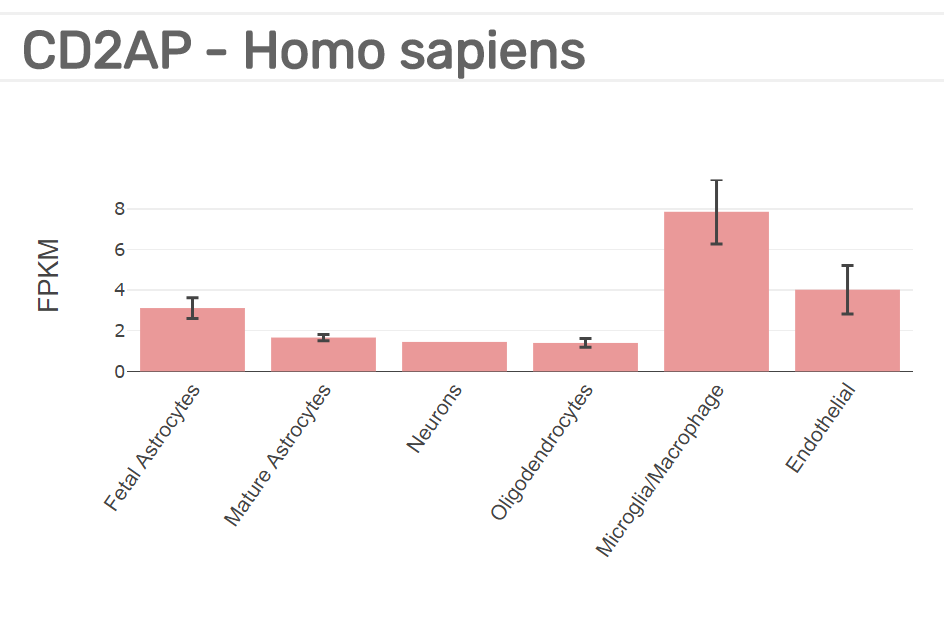


(V)


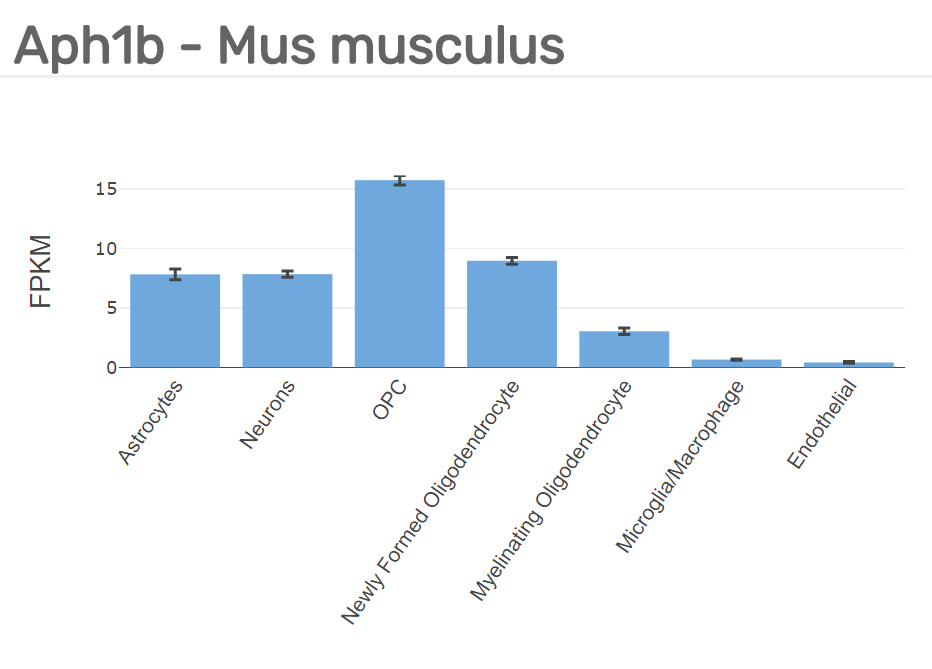


(W)


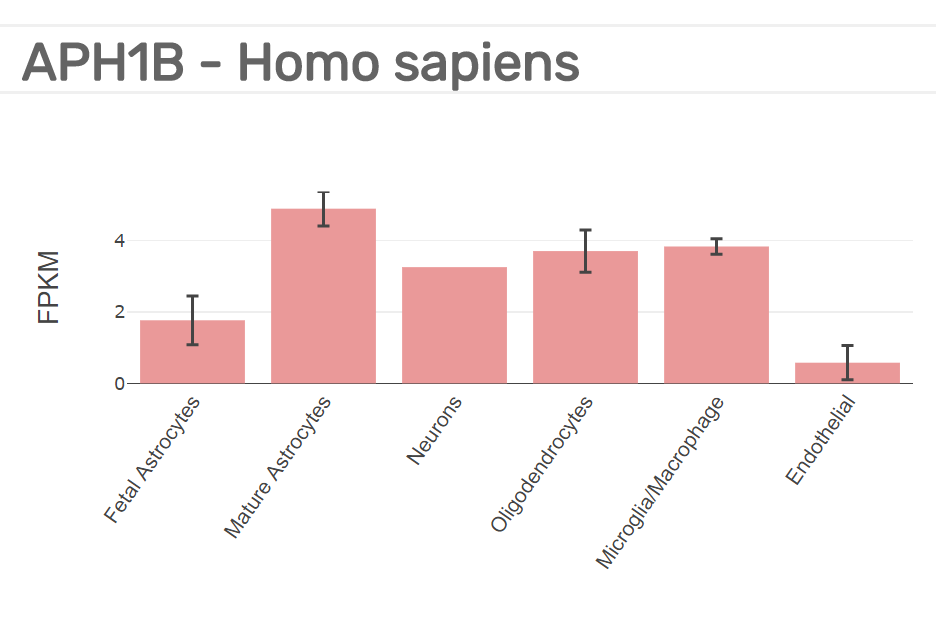

Supplement: S2 Fig — (DOCX) [file pone.0241552.s011.docx]
